# Research on Path Planning for Fire Evacuation Using the Enhanced Hiking Optimization Algorithm

**DOI:** 10.3390/biomimetics11040272

**Published:** 2026-04-15

**Authors:** Faguo Zhou, Yi Wu, Zhe You, Shuyu Yao, Kaile Lyu, Menglin Chen, Jianshen Yang

**Affiliations:** School of Artificial Intelligence, China University of Mining and Technology-Beijing, Beijing 100083, China; sqt2310405033@student.cumtb.edu.cn (Y.W.); sqt2310405037@student.cumtb.edu.cn (Z.Y.); sqt2310405036@student.cumtb.edu.cn (S.Y.); sqt2410405029@student.cumtb.edu.cn (K.L.); sqt2410405017@student.cumtb.edu.cn (M.C.); zqt2310405042@student.cumtb.edu.cn (J.Y.)

**Keywords:** Hiking Optimization Algorithm, metaheuristic algorithm, numerical optimization, fire evacuation path planning, swarm intelligence

## Abstract

To address the key challenges in fire evacuation path planning, such as the tendency to converge to local optima, unbalanced computational efficiency, and suboptimal path quality, this study proposes the enhanced Hiking Optimization Algorithm of Differentiated Weighted Dynamic (WDHOA). The WDHOA integrates a three-phase cooperative framework, incorporating dynamic grouping, hybrid search, and angle generation. Comprehensive evaluations on the CEC 2017 and CEC 2022 benchmark suites demonstrate that WDHOA significantly outperforms eight widely used algorithms, such as LSHADE, RIME, SCA in convergence accuracy, stability, and robustness, especially for high-dimensional and multimodal functions. Wilcoxon rank-sum tests and Friedman tests confirm statistical significance across most functions. Ablation experiment further verifies the effectiveness of the three enhanced strategies. When applied to fire evacuation path planning, WDHOA achieves the best solutions while satisfying all nonlinear constraints. These experiments confirm that WDHOA effectively balance optimization accuracy and practical applicability in fire evacuation path planning problems.

## 1. Introduction

With rapid advancements in science and technology, the scale and complexity of optimization problems continue to grow. Many real-world optimization tasks are characterized by multimodality, high dimensionality, numerous local optima, and strict nonlinear constraints. Representative examples include UAV path planning [1], feature selection [2], wireless sensor networks [3], and job shop scheduling [4]. Traditional optimization methods, such as Gradient Descent [5], the Newton Method [6], and Conjugate Gradient Algorithms [7], typically require precise mathematical modeling to achieve effective solutions. However, these classical techniques often exhibit low computational efficiency and high resource consumption, limiting their applicability in addressing complex modern optimization problems.

In contrast, metaheuristic algorithms (MAs) have gained widespread attention due to their flexibility, practicality, and robustness in handling such challenges [8]. Among them, nature-inspired algorithms which include Genetic Algorithms (GAs) [9], Particle Swarm Optimization (PSO) [10], and the Grey Wolf Optimizer (GWO) [11], have been extensively applied and thoroughly investigated across diverse optimization domains.

Metaheuristic algorithms are inspired by a variety of bio-inspired behavioral mechanisms observed in nature. Based on the number of candidate solutions considered during the optimization process, they can be broadly classified into single-solution-based and population-based approaches [12]. Representative single-solution-based algorithms include Simulated Annealing (SA) [13] and Tabu Search (TS) [14]. SA emulates the physical annealing process of metals, gradually reducing solution energy through a controlled temperature parameter to approach the global optimum. TS, in contrast, employs a tabu list to prevent revisiting previously explored solutions, thereby reducing the likelihood of becoming trapped in local optima. Although single-solution-based methods typically feature faster search speeds due to their focus on a single candidate solution, they are also more susceptible to local stagnation because of their limited exploration of alternative regions in the search space.

Population-based metaheuristic algorithms, such as Genetic Algorithms (GAs) and Particle Swarm Optimization (PSO), overcome this limitation by maintaining a diverse set of solutions. GA simulates evolutionary processes such as selection, crossover, and mutation to iteratively improve a population of individuals, whereas PSO models the cooperative foraging behavior of bird flocks, enabling particles to search for optimal solutions through information sharing. Owing to their ability to explore multiple candidate solutions simultaneously, population-based algorithms generally demonstrate stronger global search capabilities. However, this advantage comes at the cost of higher computational demands, as large populations and complex interactions increase resource consumption and computational time. Metaheuristic algorithms can be categorized according to their source of inspiration into four types: evolution-based, swarm-intelligence-based, physics-based, and human-inspired-based algorithms [15].

The Hiking Optimization Algorithm (HOA) [16] is a novel metaheuristic method inspired by human hiking behavior. The algorithm models the search space of an optimization problem as mountainous terrain that hikers must traverse, where the varying slopes correspond to different regions of the solution landscape. By simulating the movement strategies hikers employ to navigate hills, mountains, and rocky surfaces, HOA searches for the global optimum through adaptive terrain exploration. Previous studies have demonstrated that HOA achieves strong global search ability and fast convergence across a variety of benchmark tests [17,18,19,20,21]. Despite these strengths, HOA still faces several limitations, including insufficient population diversity due to random initialization, an imbalance between global exploration and local exploitation, and a tendency to become trapped in local optima, especially when addressing high-dimensional optimization problems.

The No Free Lunch (NFL) theorem [22] states that no single algorithm can effectively solve all optimization problems, as each algorithm possesses its own strengths and inherent limitations. Consequently, pursuing a “universal algorithm” capable of addressing every optimization task is impractical. Instead, algorithms and optimization strategies should be selected according to the characteristics of the specific problem and the requirements of the application scenario. This principle underscores the importance of developing and studying metaheuristic algorithms. In this context, many researchers have sought to enhance the Hiking Optimization Algorithm (HOA) by addressing its limitations and adapting it for application domains. Xu et al. [23] proposed an improved Hiking Optimization Algorithm (CDHOA) that integrates good point set initialization, a Cauchy inverse cumulative distribution operator, and a random differential mutation strategy. Experimental results show that CDHOA outperforms eight comparative algorithms on the CEC2017 test suite, and statistical analysis confirms the significance of these performance gains. Wu et al. [24] developed an enhanced version of the HOA (CMOHOA) that incorporates Chebyshev chaotic mapping and adversarial learning strategies and applied it to task scheduling in cloud environments. Their results demonstrate that CMOHOA reduces total information transmission costs by 7%. Zhang et al. [25] introduced a multi-strategy enhanced Hiker Optimization Algorithm (MHOA) for engineering and numerical optimization, strengthening global search through a roulette wheel mechanism, enhancing population diversity via dual-mapping perturbation factors based on Logistic and Tent maps, and improving convergence accuracy and speed with a flexible position-update strategy. Peng et al. [26] improved HOA by incorporating differential evolution and a novel population generation method (BHHOA), providing an effective technical tool for enterprises implementing JRD strategies. Abdel-salam et al. [27] introduced four strategies (hierarchical random initialization, enhanced leader coordination, adaptive perturbation, and dynamic search) to develop an enhanced Hiking Optimization Algorithm (AEDHOA) for feature selection in skin cancer diagnosis. Although these improvements have achieved promising results across diverse application scenes, challenges remain. Issues such as the imbalance between global exploration and local exploitation and the tendency to fall into local optima persist, making it difficult for the algorithm to maintain consistently strong performance across different types of optimization problems.

In this work, we propose an enhanced Hiking Optimization Algorithm of Differentiated Weighted Dynamic (WDHOA) that integrates a dynamic grouping strategy, a hybrid search strategy, and a novel hiker-angle generation mechanism. Unlike existing improvement methods, WDHOA is developed directly on the foundation of the original HOA framework and simultaneously strengthens global exploration and local exploitation through three complementary components: (i) Dynamic grouping—sorting and categorizing individuals according to their fitness values, where poorly performing individuals rapidly align with higher-quality ones, thereby accelerating the algorithm’s convergence. (ii) Hybrid search—the introduction of a random exploration factor enhances the intrinsic search capability of the population and helps the algorithm escape local optima, particularly in the later stages of the search process. (iii) Angle generation—generating differential angles between inferior and superior individuals, where lower-performing individuals are guided to move efficiently toward the leaders, achieving a better balance between exploration and exploitation. Through the combined action of these three strategies, the WDHOA improves the convergence speed, stability, and robustness of the original HOA.

The performance of the WDHOA is evaluated using two benchmark function suites, CEC 2017 [28] and CEC 2022 [29]. By comparing WDHOA with eight mainstream algorithms in terms of accuracy, stability, and robustness, and by incorporating statistical tests alongside visual analyses, the effectiveness of the proposed improvement strategies is thoroughly validated. In addition, WDHOA is applied to a two-dimensional grid-based fire evacuation path planning task, further demonstrating its strong capability and practical applicability in real world optimization scenes.

The main contributions of this work are threefold:

1. Proposal of the enhanced Hiking Optimization Algorithm of Differentiated Weighted Dynamic (WDHOA): The WDHOA synergistically improves global exploration and local exploitation by integrating three complementary strategies—dynamic grouping, hybrid search, and angle generation.
2. Comprehensive performance validation: The algorithm is systematically evaluated on the CEC 2017 and CEC 2022 benchmark, as well as on two-dimensional grid-based fire evacuation path planning problems, demonstrating accuracy, stability, and efficiency across diverse optimization tasks.
3. Evidence of effectiveness through ablation and statistical analyses: Experimental and statistical results confirm that WDHOA significantly outperforms the original HOA, providing both practical value and methodological insights for the design and enhancement of swarm intelligence algorithms.

The proposed WDHOA is inspired by human hiking behaviors, where individuals explore complex terrains by dynamically forming groups, adjusting their direction based on observed paths, and balancing exploration with exploitation to efficiently reach their destinations. Such behavior reflects adaptive strategies observed in natural systems, aligning the algorithm with the principles of biomimetics, which aim to emulate efficient natural or biological behaviors in computational problem solving.

To further clarify the originality of the proposed method, as shown in Table 1, we emphasize that the WDHOA is fundamentally different from existing HOA variants such as CDHOA, MHOA, and AEDHOA. Prior enhancement strategies primarily focus on improving the initial population, introducing chaotic perturbation, or designing mutation-based operators. In contrast, the WDHOA establishes a new optimization framework based on three synergistic mechanisms. First, the WDHOA incorporates a differentiated dynamic grouping mechanism in which hikers are periodically regrouped based on both fitness ranking and step length evolution, rather than using static or random grouping. Second, the WDHOA employs an adaptive weighted hybrid search strategy that adjusts exploration and exploitation pressures through iteration dependent weight coefficients, which distinguishes it from chaotic mapping or perturbation driven exploration used in previous HOA variants. Third, the WDHOA introduces an angle-based directional generation operator that guides hikers using geometric angular relationships, enabling more informed search trajectories not found in the CDHOA, MHOA, or AEDHOA. These mechanisms collectively create a coherent dynamic search process that significantly enhances global exploration stability and reduces premature convergence.

## 2. An Enhanced Hiking Optimization Algorithm of Differentiated Weighted Dynamic (WDHOA)

HOA controls the iterative updates of searches between leader hikers and team members through a scanning factor and regulates hiker speed using the Tobler hiking function and terrain slope to achieve numerical optimization. For high-dimensional multimodal problems, HOA is prone to premature convergence due to insufficient local exploitation and pronounced singularities in search direction, resulting in unstable solution performance. To address these limitations, the WDHOA framework incorporates three improvement mechanisms: (i) Dynamic grouping strategy—sorting and classifying the population according to fitness values allows individuals with poorer fitness to quickly align with those with better fitness, enhancing convergence speed while maintaining diversity. (ii) Hybrid search strategy—introducing a random exploration factor strengthens the population’s intrinsic search ability, increases the diversity of search directions, and enables escaping local optima during later iterations. (iii) Angle-generation strategy—generating differential angles between individuals with poorer and better fitness guides inferior individuals to quickly catch up with leaders, balancing global exploration and local exploitation. Integrating these three strategies within a single framework improves the algorithm’s convergence speed, stability, and robustness in complex scenes.

### 2.1. Hiking Optimization Algorithm

HOA simulates hikers ascending mountains, hills, or rocks, and its overall mechanism comprises three consecutive stages: initialization, exploration, and exploitation. In the initialization stage, an initial population of hikers is generated within the search boundaries, and the optimal individual is identified as the leader. The exploration and exploitation stages are regulated by a scanning factor, aiming to balance global exploration and local exploitation.

The mathematical foundation of HOA is the well-known Tobler hiking function, proposed by Swiss American geographer and cartographer Waldo Tobler. This exponential function determines hiker speed by considering the steepness and slope of the terrain or path. The Tobler hiking function is expressed by the following equation:

(1) $$W_{i,t}=6e^{-3.5\left|S_{i,t}+0.05\right|},$$

where $W_{i,t}$ represents the speed of the hiker at iteration or time t, and $S_{i,t}$ denotes the slope of the trail or terrain. The slope $S_{i,t}$ is defined by the following equation:

(2) $$S_{i,t}=\frac{dh}{dx}=tan\theta_{i,t},$$

where dh and dx represent the differences in elevation and distance for the hiker, respectively. $\theta_{i,t}$ denotes the slope angle of the trail or terrain, ranging within $\left[0,\;50{}^{\circ}\right]$. HOA leverages both the social intelligence of hikers as a group and the individual cognitive abilities of each hiker. The updated or actual speed of a hiker is a function of the initial speed determined by the Tobler Hiking Function (THF), the position of the leading hiker, the hiker’s current position, and the scanning coefficient. Therefore, the current speed of hiker i is defined by the following equation:

(3) $$W_{i,t}=W_{i,t-1}+\gamma_{i,t}\cdot \left(\beta_{best}-\alpha_{i,t}\cdot \beta_{i,t}\right),$$

where $\gamma_{i,t}$ represents a uniformly distributed random number within the range $\left[0,\;1\right]$, $W_{i,t}$ and $W_{i,t-1}$ denote the current and initial speeds of hiker i, respectively. $\beta_{best}$ denotes the position of the leading hiker, and $\alpha_{i,t}$ is the scanning factor (SF) of hiker i, ranging within $\left[1,\;3\right]$. The SF ensures that hikers do not stray too far from the leader, allowing them to observe the leader’s direction and receive signals from the leader. By considering the hikers’ speed, the position update formula for a hiker is defined by the following equation:

(4) $$\beta_{i,t+1}=\beta_{i,t}+W_{i,t}.$$

In the various methods of metaheuristic algorithms, including the HOA, the initial setup of agents is a critical factor that significantly influences the accessibility of feasible solutions and the convergence speed. The HOA employs a random initialization strategy to determine the initial positions of its individuals. The initialization of hiker positions $\beta_{i,t}$ is defined by the upper $\phi_{j}^{2}$ and lower $\phi_{j}^{1}$ bounds of the solution space, as shown in the following equation:

(5) $$\beta_{i,t}=\phi_{j}^{1}+\delta_{j}\cdot \left(\phi_{j}^{2}-\phi_{j}^{1}\right),$$

where $\delta_{j}$ represents a uniformly distributed random number within $\left[0,\;1\right]$. $\phi_{j}^{1}$ and $\phi_{j}^{2}$ denote the lower and upper bounds of the j-th dimension of the decision variables in the optimization problem. The global exploration and local exploitation stages of the HOA are influenced by a parameter called the scanning factor (SF). When the SF range increases, the HOA tends toward the exploitation stage; conversely, reducing the SF range shifts the HOA toward the exploration stage. In addition, decreasing the range of the track slope angle θ guides the HOA toward the exploitation stage. These factors collectively affect the behavior and performance of the HOA in solving optimization problems.

Although the HOA is novel, several deficiencies remain in its algorithmic formulation. During the search process, it neglects cooperation among the hiker population and relies solely on the lead hiker to guide the team and update positions. In high-dimensional and complex solution spaces, this reliance can easily lead to premature convergence to local optima and weaken global exploration capability. In addition, the hiking terrain slope angle lacks adaptive adjustment: excessively large angles may skip potential solution regions and undermine convergence stability, whereas excessively small angles slow the optimization process and increase the risk of becoming trapped in local optima. These issues collectively reduce solution quality and weaken robustness, limiting HOA’s ability to address multimodal and constrained optimization problems.

### 2.2. Dynamic Grouping Strategy

The original HOA algorithm relies solely on the lead hiker to guide the team, neglecting cooperation among hikers. In challenging terrains, however, teamwork is often more effective, aligning more closely with real world hiking behavior. Therefore, a dynamic grouping strategy is introduced into HOA during the early stages of algorithm iteration. This strategy divides hikers into four distinct populations: excellent hikers $\beta_{better}$, moderate hikers $\beta_{normal}$, poor hikers $\beta_{worse}$, and elite hikers $\beta_{best}$. In each iteration, the hiker population is first sorted by fitness values from best to worst. The population consists of N individuals, with elite hikers being the best-performing individuals within the moderate population. Excellent hikers are randomly selected from the second-best to the $P_{1}$-th individual, poor hikers are randomly selected from individuals ranked $N-P_{1}+1$ to N, and moderate hikers are randomly selected from individuals ranked $P_{1}+1$ to $N-P_{1}$. The value of $P_{1}$ is not fixed and is updated in each iteration according to the following equation:

(6) $$P_{1}=\left\lfloor r_{1}\cdot N\right\rfloor ,$$

where $r_{1}$ is a random number between 0.1 and 0.3, and $\left\lfloor \cdot \right\rfloor$ denotes the floor function. Using this equation, the numbers of elite, excellent, moderate, and poor hikers can be determined dynamically. The core idea of the dynamic grouping strategy is that poor hikers strive to keep up, while elite and excellent hikers guide them toward the optimal solution. The distance differences among hikers are mathematically modeled as follows: the gap between elite and excellent hikers ${Gap}_{1}$, the gap between elite and moderate hikers ${Gap}_{2}$, the gap between elite and poor hikers ${Gap}_{3}$, and the gap between two randomly selected hikers ${Gap}_{4}$. The mathematical model for these distance differences is given by the following equation:

(7) $$\left\{\begin{array}{c} \begin{array}{c} {Gap}_{1}=\;\beta_{best}-\beta_{better}\\ {Gap}_{2}=\;\beta_{best}-\beta_{normal}\\ {Gap}_{3}=\;\beta_{best}-\beta_{worse}\\ {Gap}_{4}=\;\beta_{L_{1}}+\beta_{L_{2}} \end{array} \end{array}\right.,$$

where $L_{1}$ and $L_{2}$ are two distinct individuals randomly selected from the hiker population. To capture variability among different hiker populations, a learning factor (LF) is introduced for each of the four different measures. LF influences the learning behavior of individuals, and the value of ${LF}_{i}$ is determined by the following equation:

(8) $$LF_{i}=\frac{Gap_{i}}{\sum_{i=1}^{4}Gap_{i}},i=1,2,3,4.$$

From the calculation formula of ${LF}_{i}$, it can be observed that when the gap in the i-th dimension of the hiker population is larger; $LF_{i}$ also increases, allowing individuals to learn more from this gap. Different individuals respond differently to this variation. For example, individuals with high fitness values require less learning from this distance, whereas poorer-performing individuals should learn more from this difference measure.

This normalized factor ensures that larger distance differences contribute more to learning, promoting adaptive exploration along the dimensions exhibiting greater diversity. Its derivative with respect to ${Gap}_{i}$ is positive:

(9) $$\frac{{\partial LF}_{i}}{{\partial Gap}_{i}}=\frac{\sum_{j\neq i}{Gap}_{j}}{{\left(\sum_{j=1}^{4}{Gap}_{j}\right)}^{2}}>0,$$

indicating that an increase in ${Gap}_{i}$ results in proportionally larger learning influence.

To balance the learning effect of this distance difference among individuals, a differentiation factor $\rho_{i}$ is introduced to enable targeted learning within the hiker population. The value of $\rho_{i}$ is determined by the following equation:

(10) $$\rho_{i}=\frac{{fit}_{i,t}}{max\left({fit}_{i,t}\right)},$$

where ${fit}_{i,t}$ is the fitness value of the i-th individual at iteration t, and $max\left({fit}_{i,t}\right)$ is the maximum fitness value among all individuals at iteration t. It can be observed that, when an individual’s fitness value is small (indicating better performance), the differentiation factor also becomes smaller, meaning extensive learning from the difference is unnecessary. Conversely, when an individual’s fitness value is large (indicating poorer performance), the differentiation factor increases, reflecting the need for stronger learning from the difference.

For the i-th group of gaps ${Gap}_{i}$, individual i absorbs knowledge from these gaps, forming the i-th group of knowledge acquisition. This process is illustrated by the following equation:

(11) $$KA_{i}=\rho_{i}\cdot LF_{i}\cdot Gap_{i},i=1,2,3,4.$$

The partial derivatives of $KA_{i}$ with respect to ${Gap}_{i}$ and $\rho_{i}$ are strictly positive:

(12) $$\frac{{\partial KA}_{i}}{{\partial Gap}_{i}}=\rho_{i}\cdot \frac{\sum_{j\neq i}{Gap}_{j}}{{\left(\sum_{j=1}^{4}{Gap}_{j}\right)}^{2}}+\rho_{i}\cdot {LF}_{i}>0,$$

(13) $$\frac{{\partial KA}_{i}}{{\partial \rho }_{i}}={LF}_{i}\cdot {Gap}_{i}>0,$$

indicating that larger distance gaps or larger differentiation factors result in stronger updates, reinforcing adaptive exploration for individuals with higher gaps or poorer fitness.

At this stage, the hiker’s position is updated according to the following equation:

(14) $$\beta_{i}^{t+1}=\beta_{i}^{t}+\sum_{i=1}^{4}KA_{i}.$$

At early iterations, the distance differences ${Gap}_{i}$ are generally large, and the differentiation factors $\rho_{i}$ are relatively high for poorly performing individuals. Consequently, the knowledge acquisition term Equation (11) attains large values, resulting in substantial position updates Equation (14) which promotes extensive global exploration.

As iterations progress, ${Gap}_{i}$ gradually decreases and $\rho_{i}$ diminishes for most individuals, reducing $KA_{i}$ and thereby moderating the step size.

In the final stages, ${Gap}_{i}\to 0$ and $\rho_{i}\to 0$, leading to negligible updates and enabling precise local exploitation. This naturally enforces a transition from exploration to exploitation without the need for external parameter scheduling.

The differentiation factor, $\rho_{i},$ ensures that learning is tailored according to individual performance. Superior individuals with lower fitness values perform smaller updates, preserving convergence precision, while poorly performing individuals with larger fitness values undergo stronger updates, preventing premature stagnation and encouraging efficient exploration of under-explored regions.

Considering the asymptotic behavior as t→T, we observe:

(15) $$\left\{\begin{array}{c} \underset{t\to T}{\lim}{Gap}_{i}=0,\\ \underset{t\to T}{\lim}\rho_{i}=0,\\ \underset{t\to T}{\lim}{KA}_{i}=0. \end{array}\right.$$

Therefore, the position update converges to 0:

(16) $$\underset{t\to T}{\lim}\left(\beta_{i}^{t+1}-\beta_{i}^{t}\right)=0,$$

indicating that the population stabilizes around optimal solutions. This property guarantees asymptotic stability of the algorithm.

The knowledge acquisition formulation Equation (11) enables the algorithm to automatically adjust the magnitude of updates based on population diversity and individual fitness. Large gaps and poorly performing individuals induce stronger learning, while small gaps and superior individuals result in finer adjustments. This self-regulating mechanism effectively balances exploration and exploitation throughout the search process, eliminating the need for manual tuning of exploration parameters.

It can be observed that, in the HOA, introducing a dynamic grouping strategy during the early stages of the algorithm strengthens collaboration among hikers, thereby enhancing overall team coordination in the initial iterations. As a result, poorer-performing hikers quickly converge toward elite and excellent hikers, accelerating the algorithm’s convergence speed.

### 2.3. Hybrid Search Strategy

In the original HOA, the hiking leader is intended to guide the team rapidly toward the destination. However, blindly following the leader’s pace often causes individuals to lose initiative. To enable all individuals to explore randomly within their own domains and enhance global exploration, while preventing excessive deviation from the hiking leader and improving local exploitation, a random exploration factor, ω, is introduced.

Here, ω is a random number between 0 and 1. The random exploration strategy is executed only when ω < 0.5; otherwise, the original strategy remains unchanged. The local search strategy is formulated as follows:

(17) $$\beta_{i}^{t+1}=\beta_{i,j}^{t}+r\cdot \left(\beta_{j}^{best}-\beta_{i,j}^{t}\right),$$

where r is a random number between 0 and 1, and $\beta_{j}^{best}$ denotes the j-th dimension of the elite hiker. To enhance local exploitation and improve the algorithm’s ability to escape local optima, a low-probability random search is introduced during the local exploitation phase. This random search factor is related to the maximum number of fitness function evaluations (MaxFEs), and is calculated as follows:

(18) $$AF=0.01+0.09\times \left(\frac{FEs}{MaxFEs}\right),$$

where FEs represents the current number of fitness function evaluations. When a random number, $r_{2}$, between 0 and 1 is less than AF, the random search strategy is executed. The formula for this strategy is as follows:

(19) $$\beta_{j}^{t+1}=lb_{j}+r_{3}\cdot \left(ub_{j}-lb_{j}\right),$$

where $lb_{j}$ and $ub_{j}$ are the lower and upper bounds, respectively, of the j-th variable in the optimization problem, and $r_{3}$ is a random number between 0 and 1.

### 2.4. Angle-Generation Strategy

It can be observed from Equations (1) and (2) that, when the range of $\theta_{i,t}$ is $\left[0,\;50{}^{\circ}\right]$, the function plots of $S_{i,t}$ and $W_{i,t}$ can be illustrated as described here.

**Figure 1:**
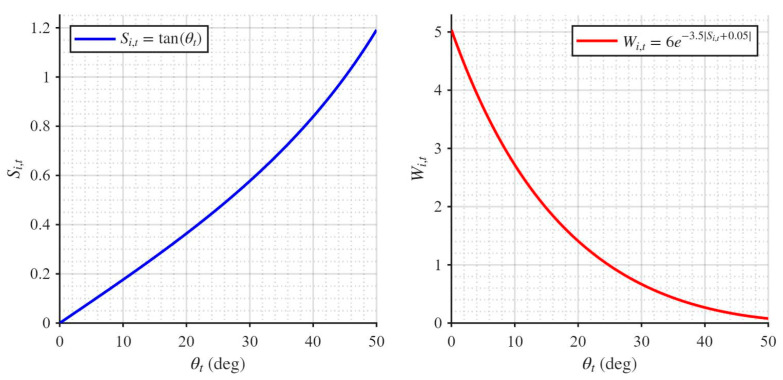
Comparison of the plots of $S_{i,t}$ and $W_{i,t}$ for $\theta_{t}\in \left[0,\;50{}^{\circ}\right]$.

It can be observed that, when $\theta_{t}\in \left[0,\;50{}^{\circ}\right]$, an increase in θ causes the growth rate of $S_{i,t}$ to gradually accelerate, and the magnitude of θ directly affects the walking speed in Tobler’s hiking function. As shown in Figure 1, when the slope angle is large, the walking speed decreases, which aligns with general walking patterns; however, this ignores individual differences among hikers. For individuals with poor fitness values, their speed should be increased to enable them to catch up with higher-fitness individuals; in this case, the slope angle should be reduced to accelerate walking speed. Conversely, for individuals with excellent fitness values, their speed should be reduced, requiring a larger slope angle. Therefore, a novel angle-generation strategy is introduced. In each iteration, the average fitness of all hikers is calculated. When an individual’s fitness value is worse than the average, it indicates that a higher speed is needed to catch up with the hiking leader, and thus the individual should explore within a smaller angle range, which is set to $\left[0,\;20{}^{\circ}\right]$. When an individual’s fitness value is better than the average, the hiker should slow down and explore within a larger angle range, which is set to $\left[20{}^{\circ},\;50{}^{\circ}\right]$. This novel angle-generation strategy better balances the algorithm’s global exploration and local exploitation capabilities.

### 2.5. Algorithm Description and Computational Complexity of WDHOA

The three strategies proposed to enhance HOA have been introduced in Section 2.2, Section 2.3 and Section 2.4. We now present the algorithmic description of WDHOA in Algorithm 1.

The computational complexity of WDHOA is $O\left(N\times D\times T\right)$, where N denotes the population size, D is the problem dimensionality, and T is the number of iterations. This complexity is identical to that of the original HOA, ensuring that WDHOA maintains practical scalability across small-, medium-, and large-scale optimization problems.

**Algorithm 1:** Algorithmic Description of the WDHOA**Input:** Maximum number of function evaluations MaxFEs, hiker population size N, dimensionality Dim, and allowable search boundaries $\left[LB,\;UB\right]$.
**Output:** Optimal solution $\beta_{best}$ and its corresponding fitness value $F_{best}$.
**1** Initialize the population using a random strategy: $X=\left[X_{1},\;X_{2},\;\ldots ,\;X_{N}\right].$
**2** Evaluate the fitness of X.
**3** Calculate the fitness of each hiker and sort them according to their fitness values, identifying the best fitness $F_{best}$ and the corresponding best position $\beta_{best}$.
**4** Let the number of iterations be t=0, set FEs=N, and define the maximum number of iterations as $MaxIter=\frac{MaxFEs}{N}$.
**5 while**
FEs≤MaxFEs && t≤MaxIter **do**
**6**  Determine the hiker with the best fitness $F_{best,t}$.
**7**  Extract the position $\beta_{best}$ corresponding to the best fitness $F_{best,t}$.
**8**  Determine $\beta_{best}$, $\beta_{better}$, $\beta_{normal}$, $\beta_{worse}$, $L_{1}$, $L_{2}$, and $max\left({fit}_{i,t}\right)$ using Equation (6).
**9  for**
i=1 to N **do**
**10**    Calculate the distance ${Gap}_{i}$ using Equation (7).
**11**    Calculate the learning factor ${LF}_{i}$ using Equation (8).
**12**    Calculate the differentiation factor $\rho_{i}$ using Equation (10).
**13**    Calculate the knowledge acquisition ${KA}_{i}$ using Equation (11).
**14**    Update the position $\beta_{i}^{t+1}$ of hiker i using Equation (14).
**15**    Calculate the average fitness ${Avg}_{fit}$.
**16**    Determine the random exploration factor ω and calculate the random search factor AF using Equation (18).
**17    if**
ω<0.5 **then**
**18**     Update the position $\beta_{i}^{t+1}$ of hiker i using Equation (17).
**19     if**
rand<AF **then**
**20**      Update the position $\beta_{i}^{t+1}$ of hiker i using Equation (19).
**21     end**
**22    else**
**23     if**
$fitness>{Avg}_{fit}$ **then**
**24**      Determine the trail elevation angle $\theta_{i,t}\in \left[0,\;20{}^{\circ}\right]$.
**25     else**
**26**      Determine the trail elevation angle $\theta_{i,t}\in \left[20{}^{\circ},\;50{}^{\circ}\right]$.
**27     end**
**28**     Calculate the slope using Equation (2)
**29**     Calculate the hiking velocity $W_{i,t-1}$ using Equation (1).
**30**     Update the actual velocity $W_{i,t}$ of hiker i using Equation (3).
**31**     Update the hiker’s position $\beta_{i,t+1}$ using Equation (4).
**32    end**
**33**    Check the boundaries and evaluate the fitness.
**34**    FEs=FEs+1.
**35**    Update FEs, $\beta_{best}$, and $F_{best}$.
**36  end**
**37**  t=t+1.
**38 end**

## 3. Experimental Comparison and Result Analysis

### 3.1. Experimental Setup

To comprehensively evaluate the WDHOA, this study employs two widely used benchmark suites: CEC 2017 and CEC 2022. Both originate from the IEEE CEC single-objective optimization competitions and have become de facto standards in evolutionary computation research. The CEC 2017 suite consists of unimodal, multimodal, hybrid, and composite functions with scalable dimensionality, making it suitable for assessing an algorithm’s balance between exploration and exploitation. Function F2 is excluded from the benchmark set due to its unstable behavior. In total, 29 single-objective benchmark functions are used for evaluation: F1 and F3 are unimodal functions; F4–F10 are simple multimodal functions; F11–F20 are hybrid functions; and F21–F30 are composite functions. These functions incorporate rotation, shifting, and other transformations to prevent overfitting. Test dimensions 10, 30, 50 and 100 are used, where higher dimensionality increases problem difficulty and computational cost.

The CEC 2022 benchmark suite presents more complex and rugged fitness landscapes characterized by numerous local optima, hybrid structures, and strong variable dependencies, enabling a more stringent assessment of an algorithm’s robustness and adaptability. A total of 12 single-objective benchmark functions is used for evaluation. These functions span several categories, including unimodal functions, basic functions, hybrid functions, and composite functions. The test dimensions considered in this study are 10 and 20. The unimodal and basic functions, such as the Zakharov, Rosenbrock, and Schaffer functions, represent classical optimization problems commonly used to evaluate the fundamental convergence behavior of optimization algorithms. Hybrid functions, constructed by combining multiple basic functions, partition variables into several subcomponents and integrate heterogeneous landscape characteristics, thereby increasing problem difficulty. Composite functions further integrate multiple basic functions into layered structures, creating highly intricate search spaces that emulate real world optimization challenges. Each function in the suite possesses distinct structural properties. For example, the Zakharov and Rosenbrock functions are unimodal and mainly used to examine convergence performance, whereas the Rastrigin and Levy functions are well-known multimodal functions suitable for evaluating global search capability. Additionally, hybrid and composite functions introduce features such as non-separability, asymmetry, and irregular variable interactions, which are intended to rigorously test the adaptability of optimization algorithms in complex environments.

Using these two benchmark suites jointly offers a comprehensive and complementary perspective on algorithmic performance across heterogeneous problem characteristics and varying levels of difficulty. Together, they enable a balanced evaluation of global exploration, local exploitation, robustness, and adaptability under diverse landscape structures. The summary of the CEC 2017 and CEC 2022 benchmark are provided in Table 2 and Table 3.

To ensure a fair and consistent comparison, all experiments in this study were conducted under a unified computational environment. The numerical evaluations were performed on a machine running the Windows 10 operating system. The hardware configuration included an Intel Core i9-10980 CPU, an NVIDIA RTX 3090 GPU with 16 GB of video memory, and 256 GB of RAM.

In addition to the original HOA algorithm, this study incorporates seven widely recognized algorithms for comparative analysis in Table 4. These comparison methods are categorized as follows: LSHADE [30], an algorithm that has demonstrated champion-level performance on CEC 2014 benchmark; SFOA [31], STOA [32], SCA [33], RIME [34], and KOA [35], which are five highly cited metaheuristic algorithms proposed in recent years; and CPSOGSA [36], a hybrid optimization method that integrates multiple enhancement strategies to improve performance.

Comparative experiments are conducted using the 20 dimensions CEC 2022 benchmark and the 10, 30, 50 and 100 dimensions CEC 2017 benchmark, while ablation experiment employed the 10 dimensions CEC 2022 benchmark. To ensure consistency and fairness across both comparative and ablation studies, and to mitigate the impact of stochastic variations in single runs, the stopping criterion is standardized based on the maximum number of fitness function evaluations. Specifically, all selected algorithms are evaluated under a unified experimental setting: population size of N=30, a maximum of MaxFEs=30,000, with each test function independently executed 30 times. The evaluation metrics included the standard deviation (Std.), average fitness value (Avg.), and average fitness rank (Rank). Overall algorithm performance is visualized using radar charts, average fitness rank plots, and box plots.

### 3.2. Results for 20 Dimensions on the CEC 2022 Benchmark

Table 5 summarizes the numerical optimization results of the WDHOA, HOA, and seven additional comparison algorithms for 20 dimensions on the CEC2022 benchmark, comprising a total of 12 test functions. For each function, the best average fitness value and the best standard deviation are highlighted in bold. The experimental results are analyzed according to the optimization performance on different types of test functions.

F1, F2, and F3 are unimodal functions with a single global optimum, primarily used to evaluate algorithm convergence speed. F4 and F5 are multimodal functions with multiple local optima, which test the ability of algorithms to escape local minima. As shown in Table 3, the WDHOA significantly outperforms the original HOA on all five basic functions, achieving leading average fitness ranks and favorable standard deviations. Compared with the LSHADE champion algorithm, the WDHOA ranks first among four functions and is slightly inferior to F4, where it ranks third. These results indicate that the WDHOA provides stable and robust performance on basic functions, with minimal fluctuations in both average fitness and standard deviation.

F6, F7 and F8 are designed to test the global exploration capability of algorithms. WDHOA achieves the best on F6 and F8, while on F7 it attains the smallest standard deviation, with an average fitness value of $2.038\times 10^{3}$, closely matching LSHADE’s $2.034\times 10^{3}$. Compared to the HOA and other highly cited metaheuristic and hybrid algorithms, the WDHOA and LSHADE consistently occupy the top ranks, with the WDHOA securing two first rankings and one first standard deviation, whereas LSHADE obtains the best average fitness on F7. Notably, the original HOA, SFOA, STOA, SCA, and KOA exhibit unstable fluctuations on F6, whereas the WDHOA maintains consistent results, further demonstrating its stability. These findings indicate that the WDHOA possesses strong global exploration capabilities and excels in solving hybrid optimization problems.

F9, F10, F11 and F12 involve more complex scenes, including non-separability and asymmetry, and are intended to test algorithm adaptability in challenging environments. The WDHOA ranks first on F9 and F12, while LSHADE achieves the best on F10 and F11, with the WDHOA achieving second place. Examining the standard deviations, the WDHOA’s values for these four functions are $2.801\times 10^{-13}$, 145.5, 25.37, and 5.851, respectively, indicating minimal fluctuations in average fitness values. This demonstrates that the WDHOA maintains adaptability in complex optimization environments, achieving optimal or near-optimal solutions comparable to champion algorithms, without being adversely affected by problem complexity.

**Figure 2:**
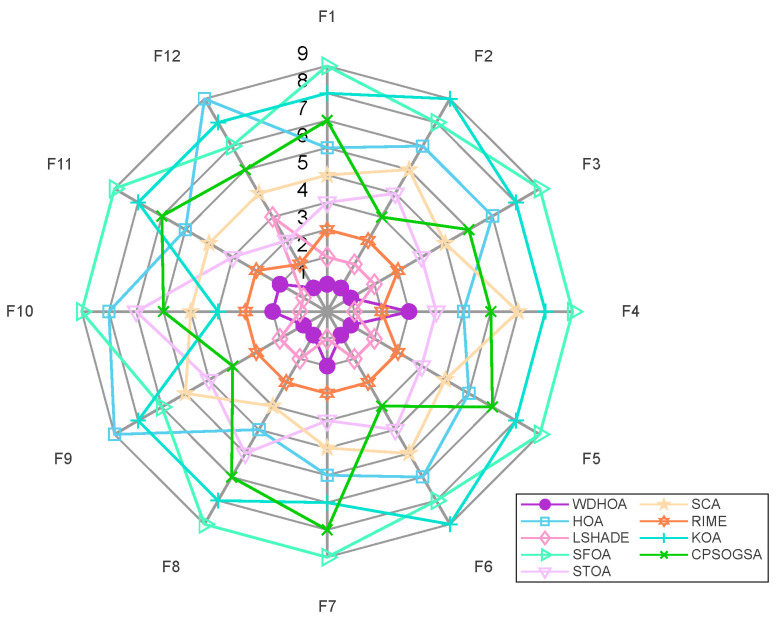
Radar map for 20 dimensions on the CEC 2022 benchmark.

**Figure 3:**
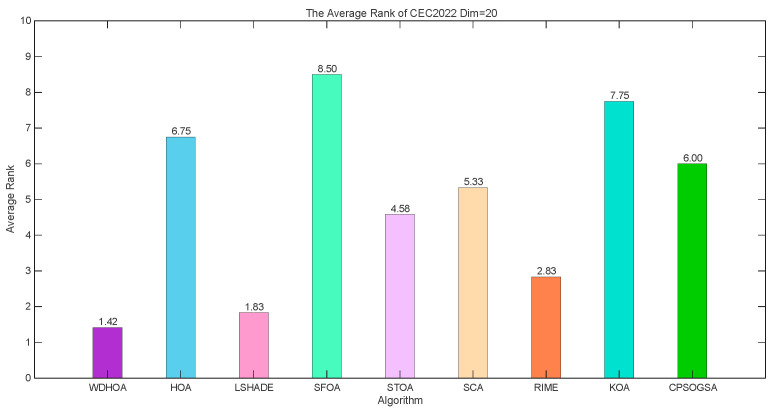
Average rank plot for 20 dimensions on the CEC 2022 benchmark.

Based on the experimental results in Table 5, radar charts and average fitness rank plots for 20 Dimensions on the CEC 2022 Benchmark are generated, as shown in Figure 2 and Figure 3. From the radar chart in Figure 2, the thick purple circular markers indicate that the WDHOA achieved first place on eight functions: F1, F2, F3, F5, F6, F8, F9, and F12. The WDHOA ranked second on three functions (F7, F10, F11) and only ranked third on F4. In comparison, the LSHADE champion algorithm obtained first place on only four functions: F4, F7, F10, and F11.

Further inspection reveals that the closed shape formed by the blue solid square markers representing the original HOA is skewed outward, whereas the shape corresponding to the WDHOA is skewed inward. Moreover, the area enclosed by the WDHOA’s radar plot is the smallest among all nine algorithms. A comprehensive analysis indicates that, on the CEC 2022 benchmark, the WDHOA demonstrates excellent and stable performance, with minimal fluctuation in average fitness rank across all test functions. Across the majority of unimodal, multimodal, hybrid, and composite functions, the WDHOA consistently achieves first place in providing optimal solutions.

From the average fitness rank plot in Figure 3, it is evident that the WDHOA achieved an average fitness rank of 1.42 across the 12 test functions, ranking first among the nine comparison algorithms. The subsequent rankings were LSHADE (1.83), RIME (2.83), STOA (4.58), SCA (5.33), CPSOGSA (6.00), HOA (6.75), KOA (7.75), and SFOA (8.50). Based on these average fitness ranks, the WDHOA, LSHADE, and RIME are the three best-performing algorithms, with the lowest (i.e., best) average ranks. While the original HOA exhibited a relatively poor average rank, the improvements introduced in the WDHOA increased its average rank significantly from 6.75 to 1.42, surpassing the LSHADE champion algorithm’s average rank of 1.83. This analysis indicates that the WDHOA demonstrates excellent overall solution performance, outperforming all other comparison algorithms in terms of average fitness ranking.

**Figure 4:**
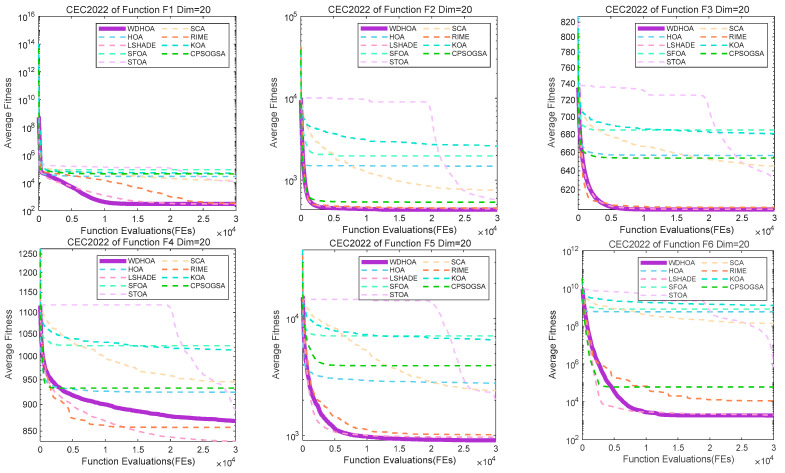
Convergence curves for 20 dimensions on the CEC 2022 benchmark (F1–F6).

The six convergence curves in Figure 4 correspond to F1–F3 (unimodal functions), F4 and F5 (multimodal functions), and F6 (a hybrid function). From these plots, it can be observed that the WDHOA exhibits rapid convergence. For instance, on F1, the convergence curve stabilizes after approximately 10,000 fitness function evaluations. On F2, F3, and F5, WDHOA’s curves are positioned lower than those of most comparison algorithms, indicating efficient exploration from the initial conditions and demonstrating strong competitive performance. For the multimodal function F4, although WDHOA’s convergence speed and solution accuracy are slightly behind LSHADE and RIME, it still significantly outperforms the remaining comparison algorithms, highlighting its robustness in multimodal landscapes.

**Figure 5:**
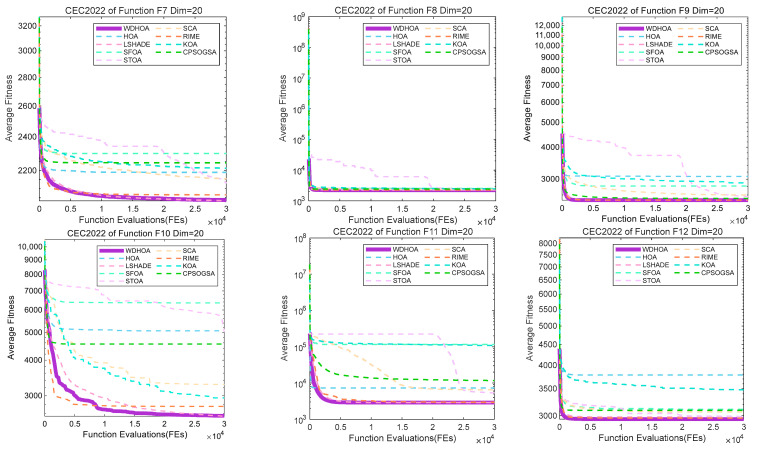
Convergence curves for 20 dimensions on the CEC 2022 benchmark (F7–F12).

**Figure 6:**
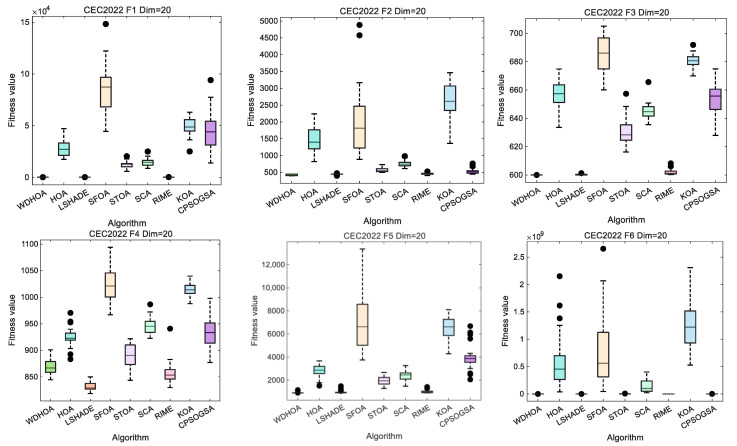
Box plots for 20 dimensions on the CEC 2022 benchmark (F1–F6).

The convergence curves presented in Figure 5 correspond to F7 and F8 (hybrid functions) and F9–F12 (composition functions). From Figure 6, it can be observed that even when facing more complex and challenging optimization problems, WDHOA maintains stable performance, with its convergence curves consistently positioned lower than those of most comparison algorithms. For F8, F9, and F12, WDHOA rapidly converges during the early stages of fitness function evaluations, demonstrating a clear advantage in convergence speed. On F10, the convergence curve exhibits a brief plateau before declining and gradually stabilizing, although its final convergence speed is slightly slower than that of LSHADE, WDHOA still achieves the second-place solution, reflecting a strong ability to escape local optima and continue effective search. For the composite functions F11 and F12, several comparison algorithms, such as CPSOGSA and RIME, produce poor-quality solutions during the initial evaluation stages, with fitness values at a higher order of magnitude, whereas WDHOA maintains a stable and effective initial search capability. Overall, analysis of the convergence curves across all 12 test functions indicates that WDHOA converges rapidly, exhibits strong capability to escape local optima, and demonstrates superior overall performance compared to the other algorithms when addressing optimization problems of varying complexity.

Box plots provide a visual representation of the deviation of each algorithm in locating optimal solutions across different test functions, intuitively reflecting the fluctuation and stability of the results. Box plots are generated for all 12 test functions, with those corresponding to F1–F6 presented in Figure 6.

Analysis of the box plots for F1–F6 as shown in Figure 6, which indicates that except for a slight deviation in WDHOA’s solution on F4, the fluctuation levels on the remaining five functions are substantially lower than those of the comparison algorithms. A detailed examination reveals that SFOA exhibits the largest solution fluctuations, followed by the original HOA algorithm. For the LSHADE champion algorithm, minor fluctuations are also observed on F4, while its performance on the other five functions remains stable. Overall, these results demonstrate that WDHOA maintains excellent solution stability on both unimodal and multimodal functions, with minimal deviation when locating optimal solutions.

**Figure 7:**
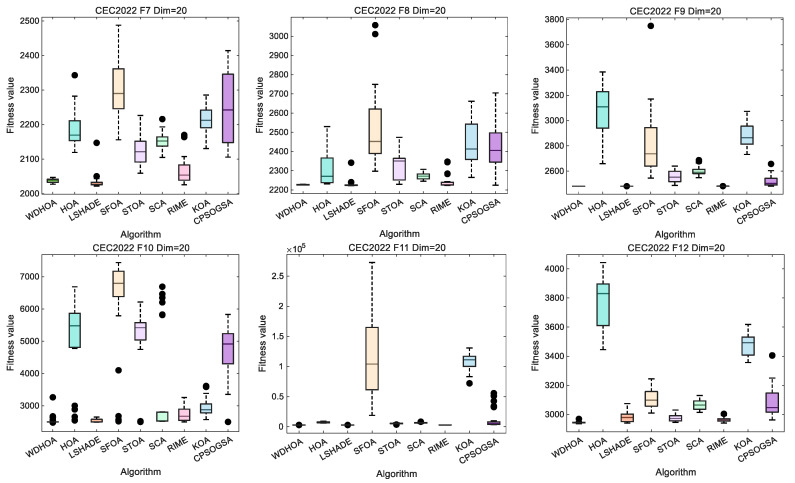
Box plots for 20 dimensions on the CEC 2022 benchmark (F7–F12).

Analysis of the box plots for F7–F12 as shown in Figure 7, which indicates that, on these more challenging hybrid and composite functions, WDHOA exhibits minimal solution deviation and fluctuation, clearly outperforming the original HOA algorithm. Specifically, for F8, all eight comparison algorithms display varying degrees of solution fluctuation, whereas WDHOA maintains consistently stable performance. On F12, LSHADE, RIME, and several other highly cited hybrid algorithms show pronounced fluctuations in their box plots, yet WDHOA continues to demonstrate strong stability. Overall, the box plot analysis across all 12 test functions confirms that WDHOA provides high solution stability, minimal fluctuation, and a significant competitive advantage over existing algorithms.

The Wilcoxon rank-sum test results presented in Table 6 show that, at the significance level of α=0.05, the WDHOA achieves statistically significant differences compared with many conventional algorithms. Notably, relative to the original HOA, the WDHOA attains significant improvements across all CEC 2022 benchmarks. These results further confirm that the three enhancement strategies effectively accelerate convergence while maintaining a robust balance between global exploration and local exploitation.

### 3.3. Results for 10, 30, 50 and 100 Dimensions on the CEC 2017 Benchmark

High-dimensional optimization problems have long presented substantial challenges in the optimization community, and evaluating an algorithm’s performance on such problems is essential for assessing its generalization capability and robustness. High-dimensional tests also offer a more comprehensive examination of an algorithm’s solution accuracy and computational efficiency when confronted with complex and difficult optimization tasks. In this section, comparative experiments are conducted for 10, 30, 50 and 100 dimensions on the CEC 2017 benchmark.

From Table 7, Table 8, Table 9 and Table 10 for unimodal functions, WDHOA achieves the top rank among all comparison algorithms on F1 and F3. For F1, the other eight algorithms exhibit extreme abnormal values during optimization, with average fitness values and standard deviations reaching magnitudes of $10^{8}$ or higher. In contrast, WDHOA maintains a fitness magnitude of $10^{3}$, demonstrating its stable optimization capability on high-dimensional unimodal problems. For F3, WDHOA again obtains both the best standard deviation and the lowest average fitness value, outperforming all competing algorithms.

For multimodal functions F4–F10, the WDHOA achieves the best average fitness performance on F4, F6, and F9. Although it does not obtain first place on the remaining four multimodal functions, the WDHOA still delivers high accuracy solutions. For example, on F7, the WDHOA ranks second with an average fitness of $1.126\times 10^{3}$, slightly worse than RIME’s $1.043\times 10^{3}$, yet the WDHOA achieves the best standard deviation 46.31, indicating strong solution stability. On F8, the WDHOA ranks third in average fitness, but its result of $1.124\times 10^{3}$ is close to RIME’s optimal value $1.025\times 10^{3}$, and the standard deviation values further support this observation. Overall, the WDHOA remains highly competitive on multimodal functions.

For hybrid functions F11–F20, the WDHOA achieves first place performance on seven hybrid functions: F11, F12, F13, F14, F15, F18, and F19. In comparison, the high-performance champion algorithm LSHADE ranks first only on F16, F17, and F20. The original HOA suffers from extremely large abnormal values on several hybrid functions, and other algorithms show similar instability. For instance, on F13, all algorithms except the WDHOA, LSHADE, and RIME produce solutions at magnitudes of $10^{9}$ or higher, indicating poor optimization performance. RIME achieves a magnitude of $10^{5}$, LSHADE achieves $10^{4}$, while the WDHOA reaches only $10^{3}$, demonstrating the highest accuracy, the smallest fluctuation, and overall superiority. The WDHOA also shows noticeably better performance across the remaining hybrid functions, highlighting its strong optimization ability and stability in complex high-dimensional scenes.

For composition functions F21–F30, the WDHOA achieves first place rankings on seven composite test functions as well, clearly outperforming both the original HOA and the high performance LSHADE algorithm. Moreover, the WDHOA maintains notable stability and robustness throughout the optimization process on composite functions. For example, on F30, all comparison algorithms, except the WDHOA and LSHADE, exhibited severe fluctuations in both the average fitness and standard deviation, indicating difficulty in handling extremely challenging high-dimensional composite problems. In contrast, the WDHOA remains highly stable under these demanding conditions. The ten composite test functions further demonstrate the WDHOA’s strong optimization capability and excellent robustness when facing complex and difficult optimization tasks.

**Figure 8:**
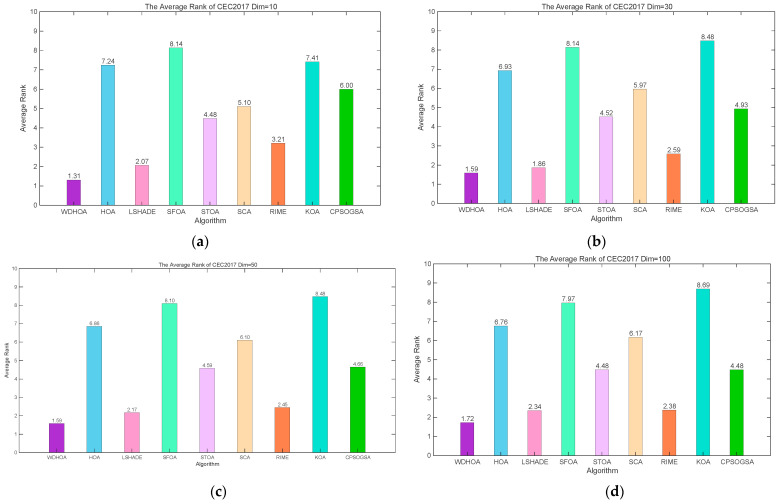
Average rank plot for 10, 30, 50 and 100 dimensions on the CEC 2017 benchmark: (**a**) 10 dimensions; (**b**) 30 dimensions; (**c**) 50 dimensions; (**d**) 100 dimensions.

Analysis of the average fitness ranking plot in Figure 8 further supports this conclusion. The WDHOA attains the highest overall rank, with average ranking score of 1.31, 1.59, 1.59, 1.72 at 10, 30, 50 and 100 dimensions on the CEC 2017 benchmark.

**Figure 9:**
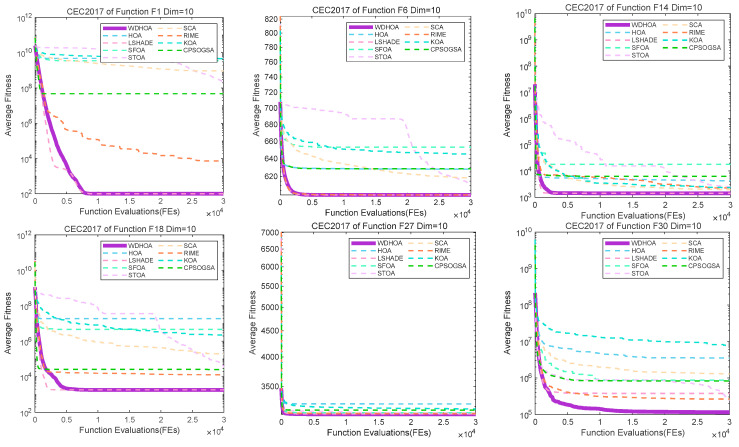
Convergence curves for 10 dimensions on the CEC 2017 benchmark (F1, F6, F14, F18, F27, F30).

**Figure 10:**
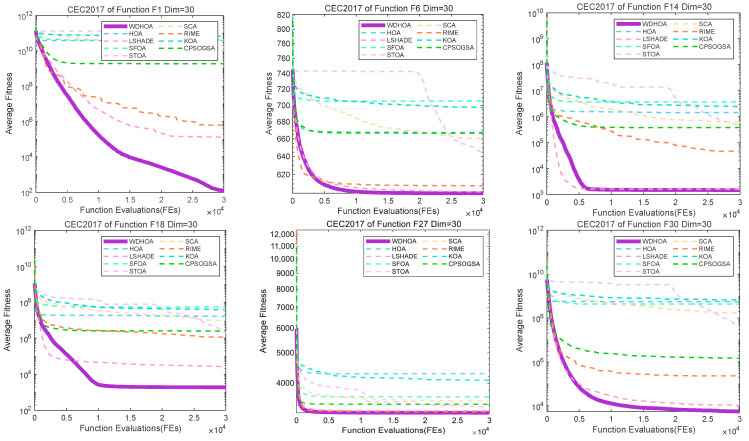
Convergence curves for 30 dimensions on the CEC 2017 benchmark (F1, F6, F14, F18, F27, F30).

**Figure 11:**
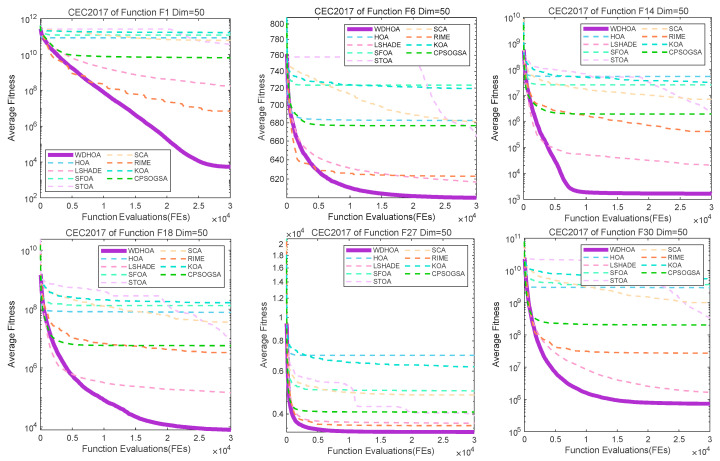
Convergence curves for 50 dimensions on the CEC 2017 benchmark (F1, F6, F14, F18, F27, F30).

**Figure 12:**
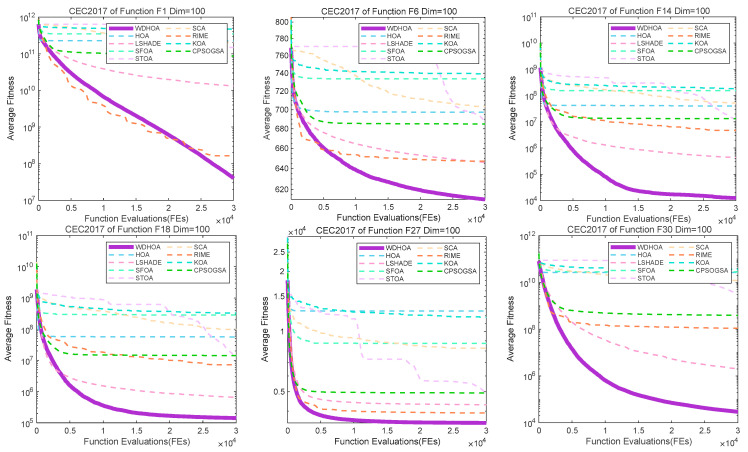
Convergence curves for 100 dimensions on the CEC 2017 benchmark (F1, F6, F14, F18, F27, F30).

To further illustrate and analyze the algorithmic performance, convergence curves of representative test functions from different categories are selected, as shown in Figure 9, Figure 10, Figure 11 and Figure 12. For the unimodal function F1, the multimodal function F6, the hybrid functions F14 and F18, and the composition functions F27 and F30, the convergence curves of the WDHOA consistently appear in the lower region of each plot. The WDHOA demonstrates a rapid reduction in average fitness value during the early stages of the search process, clearly outperforming the original HOA, the champion algorithm LSHADE, and the other comparison algorithms. As the number of fitness evaluations increases, the convergence curves progressively stabilize, further highlighting the WDHOA’s strong optimization capability and convergence reliability.

By synthesizing the evidence from the comparison tables, average fitness ranking plots, and convergence curves, it is evident that the WDHOA exhibits superior overall performance on 29 of the 10, 30, 50 and 100 dimensions on the CEC 2017 benchmark. The algorithm shows strong generalization ability and robustness across unimodal, multimodal, hybrid, and composition optimization problems. Its comprehensive performance consistently surpasses that of all competing algorithms, demonstrating a clear and substantial performance advantage.

### 3.4. Ablation Experiment

To further verify the effectiveness of the three enhanced strategies proposed for the original HOA algorithm and to clarify the specific contributions of each strategy, ablation experiment is conducted for 10 dimensions on the CEC 2022 benchmark. The algorithms included in this study are the WDHOA, the original HOA, and six additional HOA variants integrating one or two of the proposed strategies. These variants are denoted as AHOA, BHOA, CHOA, DHOA, EHOA, and FHOA. Each variant incorporates a specific combination of the dynamic grouping strategy, hybrid search strategy, and angle-generation strategy, allowing a systematic evaluation of the individual and combined effects of these enhancement components. Detailed configurations of the HOA variants are summarized in Table 11.

**Figure 13:**
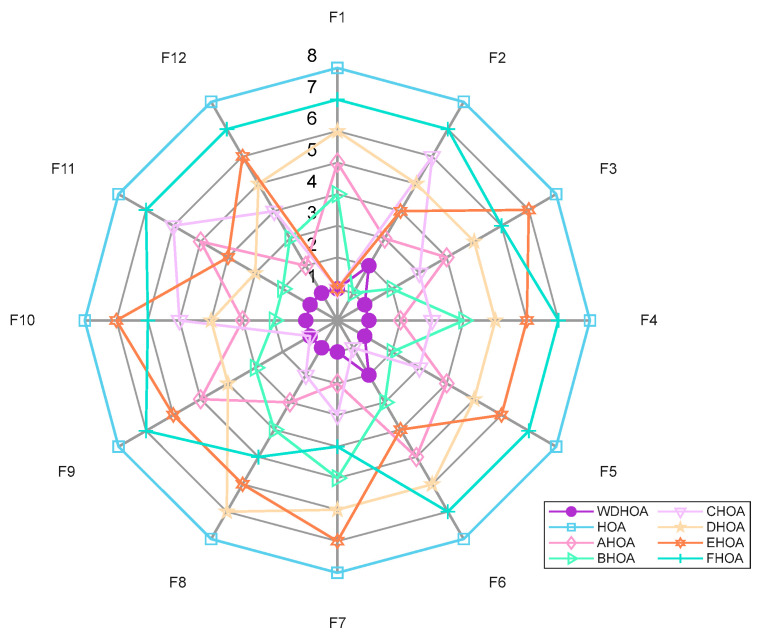
Radar map for ablation experiment on the CEC 2022 benchmark.

**Figure 14:**
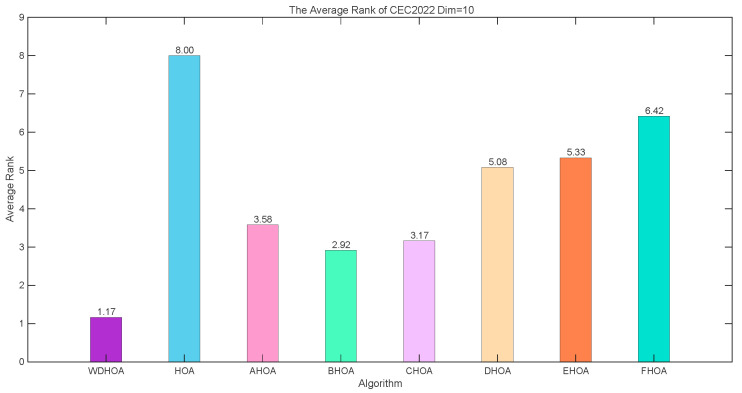
Average rank plot for ablation experiment on the CEC 2022 benchmark.

From the radar map in Figure 13 and the data in Table 12, it can be observed that the WDHOA ranks second only on F2 and F6, while achieving first place on the remaining ten test functions. The enclosed area formed by the WDHOA’s purple circular marker line is the smallest and lies closest to the center of the radar map, indicating superior overall performance. The mean fitness ranking plot in Figure 14 further confirms this observation, showing that the WDHOA achieves the best optimization performance with an average fitness rank of 1.17. The subsequent rankings are BHOA (2.92), CHOA (3.17), AHOA (3.58), DHOA (5.08), EHOA (5.23), FHOA (6.42), and HOA (8.00).

Compared with the original HOA, all variants incorporating one or more improvement strategies demonstrate enhanced performance. The WDHOA, which integrates all three strategies, outperforms the other algorithms, whereas variants combining two strategies perform better than those incorporating only a single strategy. This clearly demonstrates the effectiveness of the proposed enhancement mechanisms. The superior optimization performance of the WDHOA results from the synergistic effect of all three strategies. Among them, the angle-generation strategy contributes most significantly to performance improvement, followed by the dynamic grouping strategy, and finally the hybrid search strategy, further clarifying the relative effectiveness of each enhancement component.

### 3.5. Theoretical Proof of the Algorithm’s Global Convergence

Through the design of comparative and ablation experiments, the optimization performance of the improved WDHOA on the CEC 2017 benchmark functions at 10, 30, 50, and 100 dimensions, as well as on the CEC 2022 benchmark functions at 10 and 20 dimensions, is comprehensively evaluated, together with the effectiveness and priority of the three improvement strategies. This section theoretically demonstrates the global convergence of the WDHOA.

Suppose that, in the search space of the WDHOA, each hiker, i, at iteration t, together with all hikers, $X_{i,P_{j}}^{t}$, forms a set referred to as the search state space. The sequence composed of random variables $\left\{X_{i}^{t},\;t\geq 0\right\}$ takes discrete values over the search state space, and the iteration index t is also discretely distributed. According to the position update Equations (4), (14) and (17) in the WDHOA, it follows that in the stochastic search space, the conditional probability of the random variables satisfies:

(20) $$P\left\{X_{t+1}\left|X_{1},X_{2},\ldots ,X_{t}\right.\right\}=P\left\{X_{t+1}\left|X_{t}\right.\right\}.$$

Define the optimization problem as follows:

(21) $$\left\{\begin{array}{c} \min f\left(x\right),\\ s.t.\;\;x\in S. \end{array}\right.$$

where f:x→R denotes a real-valued function, and the set S satisfies $S=\left\{x_{min}\leq x\leq x_{max}\right\}$, where $x_{min}$ and $x_{max},\;respectively$, satisfy $x_{min}=\left({lb}_{1},{lb}_{2},\ldots ,{lb}_{d}\right),\;\;x_{max}=\left({ub}_{1},{ub}_{2},\ldots {ub}_{d}\right)$.

Let $f\left(x\right)$ be a real-valued function, where $x^{*}$ exists. If for any $x^{*}\in S$, the following holds:

(22) $$f(x^{*})\leq f(x),$$

(23) $$B^{*}=\left\{x^{*}\left|f(x^{*})\leq f(x)\right.\right\},$$

where $x^{*}$ is called the global optimum of f(x), and $B^{*}$ denotes the set consisting of all global optima.

If the following limit expression is satisfied:

(24) $$\underset{t\to +\infty }{\lim}P\left\{x^{t}\in B^{*}\right\}=1,$$

then the algorithm is said to converge to the global optimum with probability 1.

During each iteration and fitness function evaluation, the individuals corresponding to the current optimal fitness value and their position coordinates are stored. Moreover, in the position update processes of WDHOA’s dynamic grouping, hybrid search, and angle generation strategies, such as Equations (4), (14) and (17), which follow from Equations (23) and (24) that if WDHOA converges to the global optimum at iteration t, the optimal value at iteration t+1 will remain the global optimum. This implies that if a hiker has already converged to the global optimum at iteration t, the probability of failing to reach the global optimum at iteration t+1 is 0, that is:

(25) $$P\left\{x_{i}^{t}\notin B^{*}\left|x_{i}^{t-1}\in B^{*}\right.\right\}=0.$$

By consolidating the position update formulas for the hikers in the WDHOA, we obtain:

(26) $$\beta_{i}^{t+1}=\left\{\begin{array}{c} \beta_{i,j}^{t}+6e^{-3.5\left|S_{i,j}^{t}+0.05\right|},\\ \beta_{i,j}^{t}+\sum_{i=1}^{4}\frac{{fit}_{i,t}}{\max\left({fit}_{i,t}\right)}\cdot \frac{{Gap}_{i}}{\sum_{i=1}^{4}{Gap}_{i}}\cdot {Gap}_{i},\\ \beta_{i,j}^{t}+r\cdot \left(\beta_{i,j}^{best}-\beta_{i,j}^{t}\right). \end{array}\right.$$

In the early iterations of the WDHOA, its iterative form can be expressed as:

(27) $$P\left\{x_{i}^{t+1}=g_{i}^{t+1}\left|x_{i}^{t}=g_{i}^{t}\right.\right\}=P\left\{x_{i}^{t+1}=X_{i}^{t+1}\left|x_{i}^{t}=g_{i}^{t}\right.\right\}.$$

Since the probability of a candidate optimal solution being selected as the global optimum is $\frac{1}{m+1}$, suppose that at iteration t, a hiker in the WDHOA has fallen into a local optimum, with its position at iteration t+1 denoted as $X_{i}^{t+1}$. Because the dynamic grouping strategy involves distance metrics containing two randomly selected individuals and the hybrid search strategy includes a stochastic exploration factor, the WDHOA can, in the early iterations, maintain populations that learn search directions based on different distance measures. Furthermore, under the perturbation of the random search factor AF calculated in Equation (18), the algorithm exhibits strong global search capability during early iterations. As the number of evaluations and iterations increases, AF decreases, which endows the algorithm with strong local exploitation ability in later iterations. This adaptive adjustment of the search range based on changes in the random search factor prevents other individuals from falling into local optima simply by following the direction of the currently optimal individual. Therefore, the probability that the WDHOA ultimately converges to the global optimum is greater than 0, as expressed by the following formula:

(28) $$P\left\{x_{i}^{t}\in B^{*}\left|x_{i}^{t-1}\notin B^{*}\right.\right\}>0,\;t=1,2,\ldots$$

Suppose that the WDHOA individual i has not yet converged to the global optimum $B^{*}$ at iteration t, then, according to the law of total probability, we obtain:

(29) $$P\left\{x_{i}^{t}\notin B^{*}\right\}=P\left\{x_{i}^{t-1}\notin B^{*}\right\}\cdot P\left\{x_{i}^{t}\notin B^{*}\left|x_{i}^{t-1}\notin B^{*}\right.\right\}\;+P\left\{x_{i}^{t-1}\in B^{*}\right\}\cdot P\left\{x_{i}^{t}\notin B^{*}\left|x_{i}^{t-1}\in B^{*}\right.\right\}.$$

From Equation (25), it is known that in the second term on the right-hand side of Equation (29), the value of $P\left\{x_{i}^{t}\notin B^{*}\left|x_{i}^{t-1}\in B^{*}\right.\right\}$ is 0, substituting this result and simplifying yields:

(30) $$P\left\{x_{i}^{t}\notin B^{*}\right\}=P\left\{x_{i}^{t-1}\notin B^{*}\right\}\cdot P\left\{x_{i}^{t}\notin B^{*}\left|x_{i}^{t-1}\notin B^{*}\right.\right\},$$

Here, by the formula for complementary events, the second term in the product of Equation (30) satisfies the following equality:

(31) $$P\left\{x_{i}^{t}\notin B^{*}\left|x_{i}^{t-1}\notin B^{*}\right.\right\}=1-P\left\{x_{i}^{t}\in B^{*}\left|x_{i}^{t-1}\notin B^{*}\right.\right\}.$$

From Equation (28), it follows that the term after the minus sign on the right-hand side of Equation (31) satisfies $0<P\left\{x_{i}^{t}\in B^{*}\left|x_{i}^{t-1}\notin B^{*}\right.\right\}<1,$ substituting Equation (31) into Equation (30) yields:

(32) $$P\left\{x_{i}^{t}\notin B^{*}\right\}=P\left\{x_{i}^{t-1}\notin B^{*}\right\}\cdot \left\{1-P\left\{x_{i}^{t}\in B^{*}\left|x_{i}^{t-1}\notin B^{*}\right.\right\}\right\}.$$

In Equation (32), substituting t−1, t−2, …, 1 for t, respectively, yields:

(33) $$\left\{\begin{array}{c} P\left\{x_{i}^{t-1}\notin B^{*}\right\}=P\left\{x_{i}^{t-2}\notin B^{*}\right\}\cdot \left\{1-P\left\{x_{i}^{t-1}\in B^{*}\left|x_{i}^{t-2}\notin B^{*}\right.\right\}\right\},\\ P\left\{x_{i}^{t-2}\notin B^{*}\right\}=P\left\{x_{i}^{t-3}\notin B^{*}\right\}\cdot \left\{1-P\left\{x_{i}^{t-2}\in B^{*}\left|x_{i}^{t-3}\notin B^{*}\right.\right\}\right\},\\ \ldots \\ P\left\{x_{i}^{1}\notin B^{*}\right\}=P\left\{x_{i}^{0}\notin B^{*}\right\}\cdot \left\{1-P\left\{x_{i}^{1}\in B^{*}\left|x_{i}^{0}\notin B^{*}\right.\right\}\right\}. \end{array}\right.$$

Based on the recursive formula in Equation (33), substituting the calculated results of each term into Equation (32) and rearranging yields:

(34) $$P\left\{x_{i}^{t}\notin B^{*}\right\}=\prod_{k=1}^{t}\left\{1-P\left\{x_{i}^{k-1}\notin B^{*}\right\}\right\}.$$

In Equation (34), let $P\left\{x_{i}^{k}\in B^{*}\left|x_{i}^{k-1}\notin B^{*}\right.\right\}=\alpha ,$ and taking the limit on both sides of Equation (34) yields:

(35) $$\underset{t\to +\infty }{\lim}P\left\{x_{i}^{t}\notin B^{*}\right\}=\underset{t\to +\infty }{\lim}{\left(1-\alpha \right)}^{t}\cdot P\left\{x_{i}^{0}\notin B^{*}\right\},$$

since 0<α<1, it follows that $\underset{t\to +\infty }{\lim}{\left(1-\alpha \right)}^{t}=0$. Therefore, according to the arithmetic rules of limits, we have:

(36) $$\underset{t\to +\infty }{\lim}P\left\{x_{i}^{t}\notin B^{*}\right\}=0.$$

From the probability formula for complementary events, we obtain:

(37) $$P\left\{x_{i}^{t}\in B^{*}\right\}=1-P\left\{x_{i}^{t}\notin B^{*}\right\}.$$

Taking the limit on both sides of Equation (37) yields:

(38) $$\underset{t\to +\infty }{\lim}P\left\{x_{i}^{t}\in B^{*}\right\}=\underset{t\to +\infty }{\lim}\left\{1-P\left\{x_{i}^{t}\notin B^{*}\right\}\right\}\;=\underset{t\to +\infty }{\lim}1-\underset{t\to +\infty }{\lim}P\left\{x_{i}^{t}\notin B^{*}\right\}\;=1-0=1.$$

That is, $\underset{t\to +\infty }{\lim}P\left\{x_{i}^{t}\in B^{*}\right\}=1$. Therefore, from Equation (24), the WDHOA converges to the global optimum with probability 1.

In summary, a global convergence analysis of the WDHOA based on the Markov chain stochastic process demonstrates that it converges to the global optimum with probability 1.

### 3.6. Computational Complexity Analysis of the WDHOA

To evaluate the computational efficiency of the proposed WDHOA, we perform a detailed computational complexity analysis.

Let N denote the population size, D denote the problem dimensionality, and MaxFEs denote the maximum number of fitness function evaluations. The total number of iterations can be approximated as $T=\frac{MaxFEs}{N}$, For simplicity, we assume that evaluating the objective function has complexity O(D), which is common in continuous optimization problems.

The dynamic grouping strategy involves sorting the population according to fitness values at each iteration and partitioning the population into four groups. Sorting N individuals by fitness requires O(NlogN). Partitioning the population and computing the number of individuals in each group has complexity O(N⋅D). For each individual, the WDHOA computes four distance gaps: for N individuals, computing all gaps, learning factors, and differentiation factors requires O(N⋅D). The total complexity of computing ${KA}_{i}$ and updating positions is O(N⋅D). The hybrid search strategy includes random exploration and local exploitation, this step requires O(N⋅D). The angle-generation strategy adjusts the exploration range for each individual based on its fitness relative to the population mean, computing the angle range and updating positions has complexity O(N⋅D).

Summing the above contributions, the total complexity per iteration is

(39) $$T_{iter}=O\left(NlogN\right)+O\left(N\cdot D\right)+O\left(N\cdot D\right)+O\left(N\cdot D\right)+O\left(N\cdot D\right)+O\left(N\cdot D\right)\;=O\left(NlogN+5N\cdot D\right)\approx O\left(N\cdot D\right).$$

Assuming a total of $T=\frac{MaxFEs}{N}$ iterations, the overall computational complexity is

(40) $$T_{total}=T\cdot T_{iter}\approx O\left(N\cdot D\cdot MaxFEs\right).$$

### 3.7. Fire Evacuation Path Planning

In modern society, building interior designs are becoming increasingly diverse, with many structures featuring highly complex internal layouts to achieve aesthetic form and artistic expression. Such complexity poses significant challenges for occupant evacuation during fires. Although static safety evacuation maps are commonly posted inside buildings, fire ignition locations are inherently random and can spread rapidly. In real fire scenes, traditional static evacuation maps are insufficient to support dynamic, timely, and efficient evacuation. During a fire, occupants with limited visibility or respiratory difficulties may be unable to follow dynamically changing evacuation paths to exits, increasing the likelihood of crowding, congestion stairway stampedes, or entry into fire affected areas, which can lead to casualties and exacerbating property damage. Therefore, the integration of novel intelligent optimization algorithms for scientifically planning evacuation routes during building fires has emerged as a promising approach. Such methods are critical for reducing both human casualties and property losses in emergency scenes.

**Figure 15:**
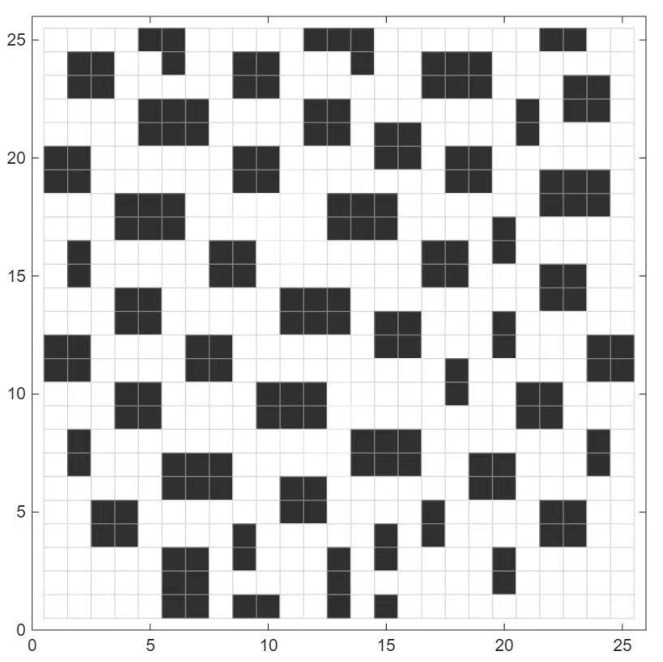
A 25 × 25 grid scene: when no fire breaks out.

When no fire breaks out, the constructed 25 × 25 grid scene is shown in Figure 15, where black indicates obstacles and white represents navigable paths.

**Figure 16:**
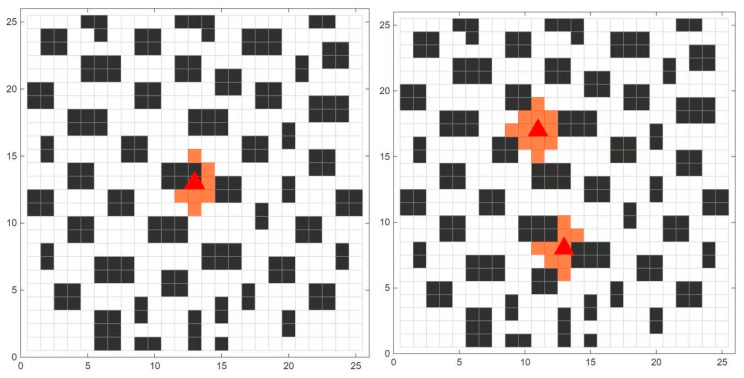
A 25 × 25 grid scene: when fire breaks out.

Considering the characteristics and locations of building fires, when fire breaks out inside the building, the evacuation scene map is shown in Figure 16, where the red triangle denotes the fire source location and the orange areas represent the grid cells affected by the fire.

The core of fire evacuation path planning lies in the formulation of an effective fitness function. To quantitatively evaluate candidate evacuation paths, five key constraints are incorporated into the mathematical model: collision cost, path length cost, heuristic cost, fire risk cost, and turning penalty cost. These components collectively capture path feasibility, efficiency, safety, and human movement characteristics. A global fitness function is then constructed using a weighted linear combination of the above constraints, enabling comprehensive assessment and optimization of evacuation routes.

The collision cost $C_{coll}$ serves as a strict boundary constraint in fire evacuation path planning. An evacuation path must not intersect black obstacle regions nor enter any grid cells affected by fires. If the trajectory violates these constraints, a penalty is imposed to ensure that the optimization algorithm avoids infeasible or unsafe regions. The collision cost is computed according to Equation (41).

(41) $$C_{coll}=\left\{\begin{array}{c} 50n,\\ when\;point\;goes\;out\;of\;bounds\;or\;collides\;with\;an\;obstacle,\\ 20n,\\ when\;point\;enters\;the\;grid\;cells\;affected\;by\;fire,\\ 0,\\ others. \end{array}\right.$$

where n represents the number of discrete points along the evacuation path whose coordinates correspond to integer grid positions.

In addition to representing the length of the evacuation path, the path length cost $C_{g}$ is defined as the sum of the Euclidean distances between all adjacent points along the path, as shown in Equation (42).

(42) $$C_{g}=\sum_{i=1}^{n-1}\sqrt{{\left(x_{i+1}-x_{i}\right)}^{2}+{\left(y_{i+1}-y_{i}\right)}^{2}},$$

where $\left(x_{i},\;y_{i}\right),\;i=1,\;2,\;\ldots ,\;n.$ represents the integer coordinate pairs of the points along the evacuation path.

To prevent the algorithm from becoming trapped in local optima and to ensure that the evacuation path ultimately reaches the target exit, the heuristic cost $C_{h}$ is introduced to accelerate convergence and guide the search direction toward the target exit, as shown in Equation (43).

(43) $$C_{h}=\sqrt{{\left(x_{end}-x_{last}\right)}^{2}+{\left(y_{end}-y_{last}\right)}^{2}},$$

where $x_{end}$ and $y_{end}$ denote the x- and y-coordinates of the target exit, $x_{last}$ and $y_{last}$ denote the x- and y-coordinates of the current path search point.

Considering human kinematic characteristics, excessive turning angles during evacuation not only reduce escape speed but may also cause falls. Therefore, a turning penalty cost $C_{turn}$ is introduced, calculated by computing the cosine of the angle between path vectors of adjacent points, as shown in Equation (44).

(44) $$cos\theta =\frac{\vec{v_{1}}\cdot \vec{v_{2}}}{\left|\vec{v_{1}}\right|\cdot \left|\vec{v_{2}}\right|},$$

when $cos\theta <\frac{\sqrt{2}}{2}$, corresponding to θ>45°, a single turning penalty is recorded, and the total number of turning penalties is denoted as $C_{turn}$.

In summary, the global fitness function $C_{total}$ is defined as a linearly weighted combination of the individual cost functions, as shown in Equation (45).

(45) $$C_{total}=\omega_{d}\cdot \left(C_{g}+C_{h}\right)+\omega_{t}\cdot C_{turn}+\omega_{c}\cdot C_{coll}.$$

The specific values of collision weight $\omega_{c}$, distance weight $\omega_{d}$,and turning penalty weight $\omega_{t}$ are set as presented in Table 13.

Minimizing the global fitness function is the objective of optimization. To ensure fairness and experimental consistency, the population size is set to N=30, the maximum number of fitness function evaluations is set to MaxFEs=30,000, and each algorithm is executed independently 30 times, with the best path selected as the result.

**Figure 17:**
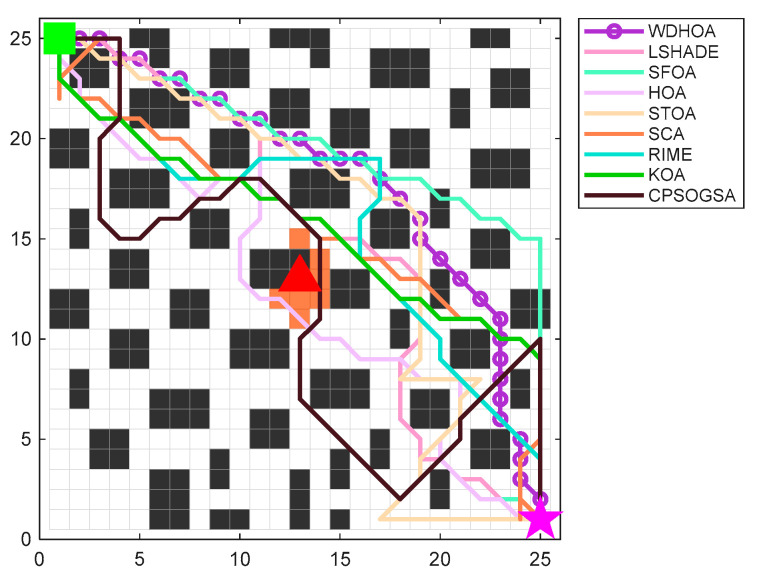
Results of single-source fire evacuation path planning.

The evacuation start point is set at (1, 25), represented by a green square, and the target exit is set at (25, 1), represented by a pink star, while the fire source is located at (13, 13). The optimal path obtained by WDHOA is depicted as a solid line with purple dots. As shown in Figure 17, the WDHOA effectively avoids collisions with obstacles and maintains a safe distance from the fire source. In contrast, the optimal path generated by the original HOA algorithm approaches the fire source near coordinates (12, 12), entering a hazardous area affected by the fire. The SFOA algorithm collides with obstacles at coordinates (25, 11) and (25, 12), while the STOA algorithm’s evacuation path encounters obstacles at multiple locations, such as (3, 24) and (7, 22). The CPSOGSA algorithm produces an excessively long path and approaches the fire source at (14, 13). The paths obtained by LSHADE and RIME exhibit excessive sharp turns, poor smoothness, and proximity to the area affected by the fire, resulting in higher risk.

**Figure 18:**
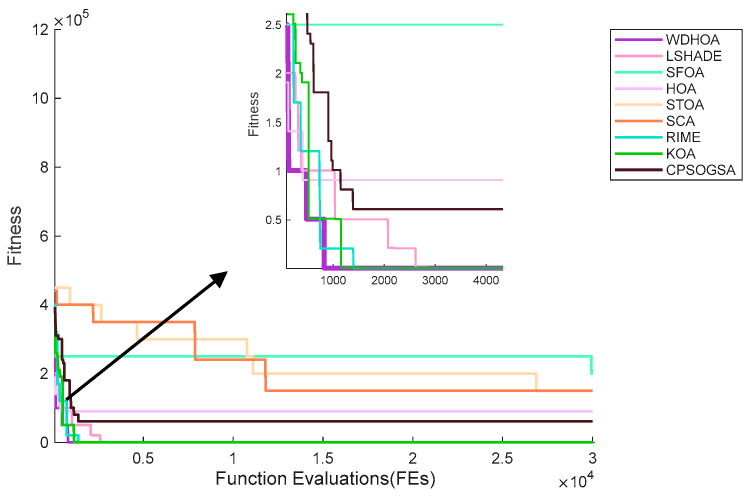
Convergence curves of single-source optimization.

As shown in Figure 18, the WDHOA rapidly converges to the optimal solution in fewer than 1000 evaluations, demonstrating the ability to escape local optima, with the subsequent convergence curve remaining stable until the 30,000th evaluation. In contrast, the convergence curve of the original HOA algorithm lies above that of WDHOA, becoming trapped in a local optimum and failing to find the global optimal path. Although LSHADE also exhibits the ability to escape local optima, its convergence rate is slower than that of WDHOA, with its curve descending below the image around 3000 evaluations. The RIME and KOA algorithms converge more slowly than WDHOA, while the SFOA, STOA, SCA, and CPSOGSA algorithms exhibit both slow convergence and entrapment in local optima during the search for the optimal evacuation path. Overall, the analysis indicates that WDHOA achieves faster convergence and consistently escapes local optima in fire breaks out, demonstrating superior optimization performance.

**Figure 19:**
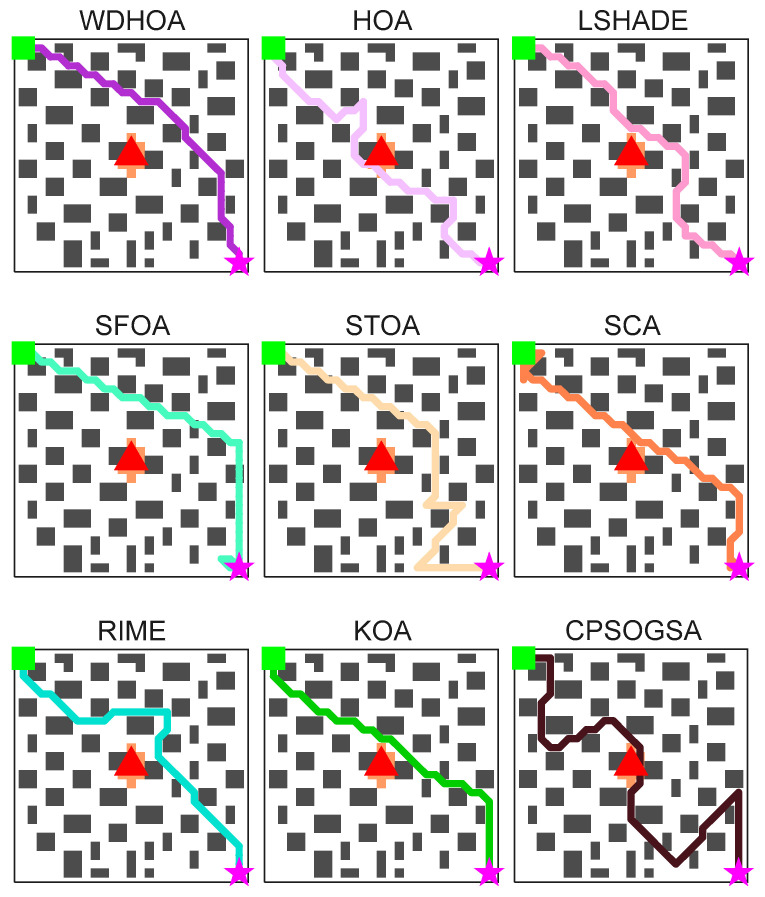
Single-source path visualization.

Table 14 provides detailed values of the global fitness function and the corresponding component costs for the nine algorithms. Based on the path visualization in Figure 19 and the data in Table 14, it is evident that WDHOA achieves the best overall performance, attaining a global fitness value of 39.21, the lowest among all algorithms. Compared with the original HOA, WDHOA yields substantial reductions in evacuation path length, fire risk cost, turning penalty, collision cost, and the global fitness value, demonstrating its superior performance. Meanwhile, Table 14 shows that the global fitness values of SFOA, STOA, and SCA are notably larger in magnitude, indicating that their optimized paths exhibit high risk. This observation is consistent with the path visualization in Figure 19, where the optimal paths generated by these three algorithms involve obstacle collisions, failing to meet evacuation safety and path feasibility requirements. Moreover, the global fitness value of WDHOA is less than that of the champion algorithm LSHADE, and its path avoids fire spread areas, excessive turning, and obstacle collisions. These results highlight the strong optimization capability of WDHOA in fire evacuation path planning, demonstrating that it can consistently deliver stable and feasible optimal paths even under complex scenes.

**Figure 20:**
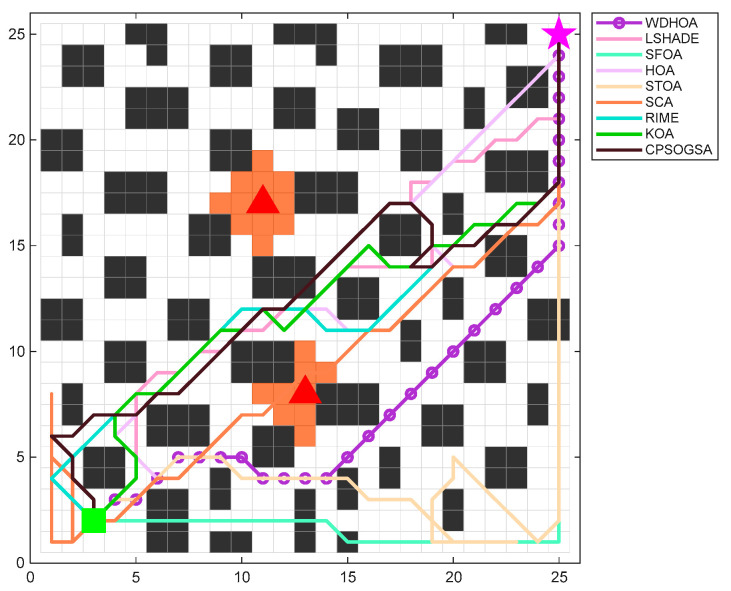
Results of multi-source fire evacuation path planning.

When fire breaks out inside a building, the rapid spread of flames and the scattering of sparks can ignite additional combustible materials, potentially leading to multiple simultaneous fire sources. Under such circumstances, it is necessary to perform multi-source evacuation path planning based on the on-site conditions to provide timely guidance and ensure personnel safety. To further verify the generalization capability of the WDHOA, the multi-source evacuation path planning experiment sets the evacuation starting point at coordinates (3, 1), represented by a green square, and the evacuation target exit at coordinates (25, 25), denoted by a pink star. The centers of the two fire sources are located at (11, 17) and (13, 8). The population size, maximum number of fitness evaluations, and the number of independent runs for each algorithm remain consistent with those used in the single-source evacuation path planning experiment. On the 25 × 25 multi-source evacuation path planning map, the optimal evacuation paths produced by each algorithm are plotted for comparison, as shown on Figure 20.

Figure 20 clearly illustrates the optimal evacuation paths generated by the WDHOA and the other eight comparative algorithms under the multi-source scene. The path produced by the WDHOA is depicted as a purple solid line with hollow circular markers. Its trajectory is smooth, free of obstacle collisions, and does not enter any orange regions affected by the fire. In contrast, the optimal paths obtained by the other eight algorithms exhibit various issues, including obstacle collisions, sharp turns, proximity to the two fire sources, and intrusion into fire-affected orange zones. For example, the optimal path produced by the LSHADE champion algorithm contains sharp turns at coordinates (18, 18) and (19, 14), resulting in poor path smoothness. Such frequent sharp turns may cause evacuees to stumble or fall, and the overall path shape and length are inferior to those of WDHOA. The original HOA algorithm generates obstacle collisions near the target exit at coordinates (23, 22) and (24, 23), violating physical constraints and rendering the planned route infeasible for evacuation applications.

**Figure 21:**
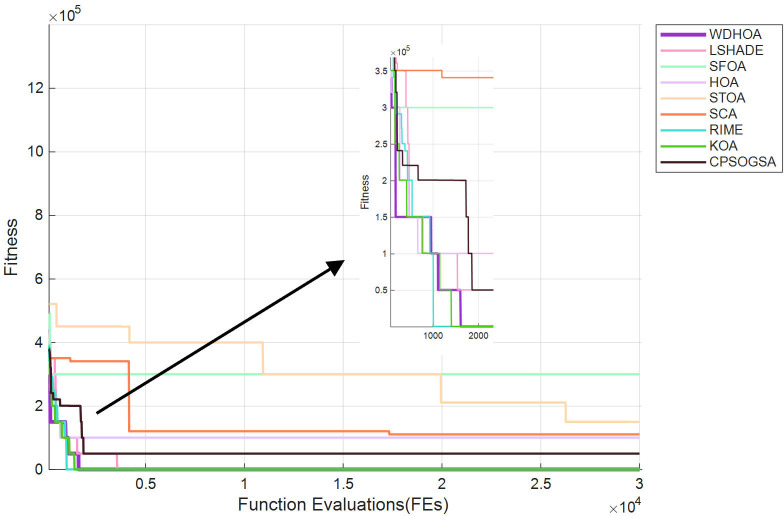
Convergence curves of multi-source optimization.

Although the evacuation path planned by the SFOA avoids the high-risk area between the two fire sources by routing from the lower part of the map, it still collides with obstacles at several locations, including (6, 2), (7, 2), (13, 2), (15, 1), (25, 11), and (25, 12). Such extensive collisions seriously violate physical constraints and make the path infeasible. The optimal path produced by the STOA not only involves obstacle collisions but also exhibits backtracking and looping near coordinate (20, 1). These behaviors are highly dangerous in actual evacuation scenarios, as backtracking and looping increase both evacuation distance and evacuation time, while also causing psychological stress on evacuees who may feel unable to locate an exit. The paths generated by the SCA, RIME, and KOA pass between the two fire sources, where the temperature and evacuation risk are higher as the fire spreads. Moreover, the optimal path planned by the SCA directly traverses the fire source center at coordinate (13, 8), posing a severe threat to evacuee safety. The convergence curves of the global fitness function with respect to the number of evaluations are plotted in Figure 21.

The convergence curves of multi-source evacuation path optimization in Figure 21 clearly illustrate the trends of the global fitness values for all algorithms as the number of evaluations increases. It can be observed that the WDHOA identifies the optimal path in fewer than 2000 fitness evaluations, after which its convergence curve becomes flat and remains stable until the end of 30,000 evaluations. Its convergence speed ranks third, behind only RIME and KOA. Although RIME and KOA converge more rapidly, the earlier analysis of the multi-source evacuation paths in Figure 18 shows that both algorithms generate suboptimal solutions, their optimal paths enter the high-risk region between the two fire sources and contain sharp turns, resulting in lower path quality compared with the WDHOA. Meanwhile, the convergence curve of the original HOA lies above that of the WDHOA, indicating that it becomes trapped in a local optimum. The LSHADE champion algorithm converges more slowly than the WDHOA, requiring approximately 5000 fitness evaluations to reach its optimum, and its path quality also remains inferior. The convergence curves of SFOA, STOA, SCA, CPSOGSA, and other algorithms lie above that of the WDHOA, indicating slower convergence and entrapment in local optima, making them unsuitable for fire evacuation path planning applications.

**Figure 22:**
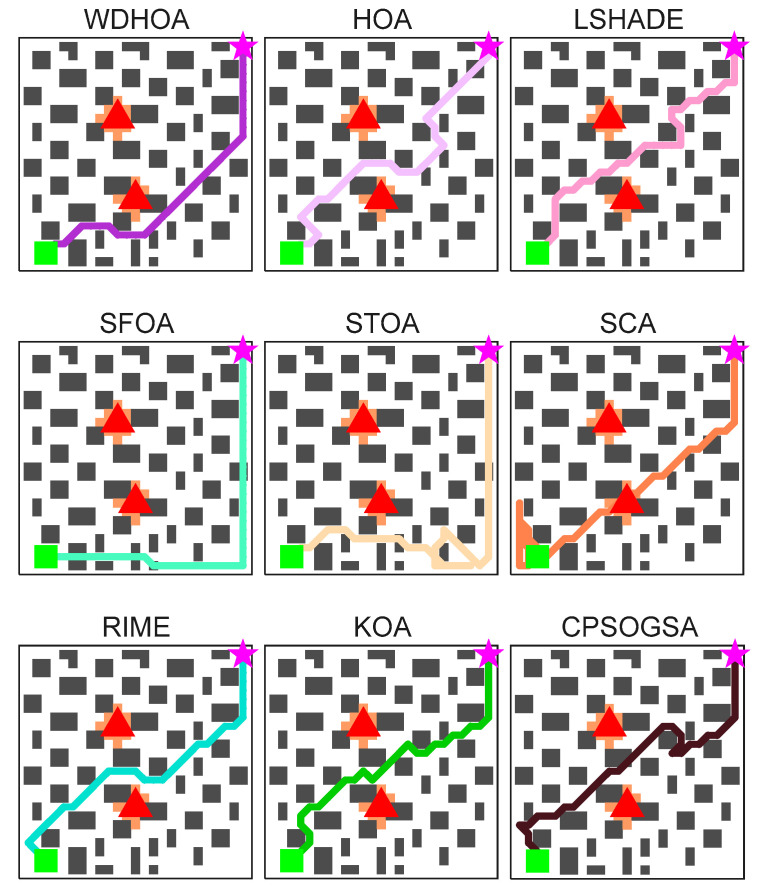
Multi-source path visualization.

The comprehensive analysis indicates that the WDHOA maintains its advantages of fast convergence and strong capability to escape local optima in multi-source evacuation path planning scene. To more intuitively illustrate the search paths of each algorithm, clearly compare the differences among their optimization trajectories, and visually quantify the optimal global fitness values achieved, visualization maps of the multi-source evacuation paths for all nine algorithms and a summary table of global fitness evaluation metrics are presented in Figure 22 and Table 15, respectively.

The evacuation path visualization results in Figure 22 presenting the optimal paths obtained by the nine algorithms. The optimal evacuation paths generated by HOA, LSHADE, RIME, KOA, and CPSOGSA all pass through the region between the two fire sources, where the ambient temperature is high and the fire continues to spread outward over time. Traversing this area inevitably exposes evacuees to significant danger. Although SFOA and STOA avoid the high-risk region between the fire sources during path planning, their evacuation paths contain obstacle collisions and instances of backtracking and looping, rendering the routes impassable during evacuation. The evacuation path planned by SCA directly crosses the orange fire-affected area, substantially increasing the risk of casualties. In contrast, the optimal path produced by the WDHOA avoids the hazardous region between the two fire sources, contains no obstacle collisions or sharp turns, and achieves the shortest overall distance. It is therefore the most desirable evacuation route in multi-fire-source scenarios and greatly enhances personnel safety.

Table 15 quantifies the strengths and weaknesses of the nine algorithms based on various cost function values and global fitness evaluations. Specifically, the optimal evacuation path identified by WDHOA has a length of 38.21 and a global fitness value of 38.21, ranking first among all algorithms. Compared with the original HOA, whose optimal path length is 41.53 and global fitness value is 100,047.53, the WDHOA demonstrates significant advantages. The optimal path obtained by the LSHADE champion algorithm contains three sharp turns, resulting in lower smoothness than the WDHOA path. Due to obstacle collisions or entry into the orange fire-risk region, the paths generated by SFOA, STOA, and SCA yield global fitness values on the order of $10^{5}$, further confirming the infeasibility of these solutions from a data-driven perspective. The evacuation paths generated by RIME and KOA still suffer from poor smoothness and excessive sharp turns, which may cause evacuees to stumble or fall during the evacuation process. Although the path produced by CPSOGSA does not enter the fire-affected orange region, it contains obstacle collisions and two sharp turns, thus failing to satisfy the absolute physical constraints and remaining unsuitable for practical evacuation.

In conclusion, the experimental results for multi-source evacuation scene demonstrate that the synergistic integration of dynamic grouping, hybrid search, and angle generation strategies in the improved WDHOA enables it to produce optimal evacuation paths that are not only the shortest but also the safest. These advantages significantly enhance personnel safety during building fire evacuations.

## 4. Discussion

The WDHOA enhances the original HOA in global exploration, local exploitation, and convergence stability through the coordinated application of dynamic grouping, hybrid search, and angle generation. Extensive experiments on CEC 2017, CEC 2022, and fire evacuation path planning consistently demonstrate improvements in solution quality, robustness, and efficiency.

Mechanistic insights: The dynamic grouping strategy ranks and partitions the population based on fitness values, enabling individuals with poorer fitness to rapidly align with superior ones, thereby accelerating convergence while preserving diversity. The hybrid search strategy introduces a random exploration factor that strengthens the population’s intrinsic search capability, increases the diversity of search directions, and facilitates escaping local optima in later iterations. The angle-generation strategy produces differential angles between inferior and superior individuals, guiding lower performing individuals to catch up with leading ones, thus balancing global exploration and local exploitation, which is a particularly advantageous feature for multimodal and high-dimensional landscapes.

Benchmark evidence: Across both benchmark suites, WDHOA achieves competitive or superior Std. and Avg. performance on most functions, indicating strong accuracy and stability. The Wilcoxon rank-sum test further corroborates these improvements, confirming their statistical reliability.

Computational considerations: Owing to the use of dynamic grouping, hybrid search, and angle generation, WDHOA incurs moderately higher computational overhead than lightweight methods such as HOA and RIME, although its overall complexity remains $O\left(N\times D\times T\right)$. When runtime is the primary concern, lightweight baselines may be more suitable; otherwise, the WDHOA’s superior accuracy and robustness justify the additional cost.

Limitations and outlook: In very high-dimensional settings, population diversity may diminish, increasing the risk of premature convergence. The current parameterization is static, which may restrict adaptability in dynamic environments. Extensions to multi-objective, dynamic, and combinatorial scenes remain to be validated. Due to limitations in experimental resources and domain-specific simulation tools, realistic fire field modeling could not be included in the present work. Therefore, the evacuation experiment is intended primarily as a proof-of-concept demonstration rather than a complete fire-safety simulation, and the influence of dynamic fire factors remains an open direction for future investigation. Future work will focus on (i) adaptive or self-tuning control of dynamic grouping, hybrid search, and angle generation; (ii) developing efficient variants for large-scale problems; and (iii) validating the method on more complex engineering tasks (e.g., multidisciplinary, heavily constrained designs and dynamic evacuation or disaster-response scenarios).

Conclusion of discussion: the WDHOA provides a principled and empirically validated enhancement to swarm optimization, improving robustness and solution quality on challenging benchmark problems and path planning task while maintaining practical scalability.

## 5. Conclusions

An enhanced Hiking Optimization Algorithm, the WDHOA, is proposed by integrating dynamic grouping, hybrid search, and angle generation strategies to balance global exploration and local exploitation. Across CEC 2017, CEC 2022, and fire evacuation path planning, the WDHOA outperforms eight representative metaheuristics in convergence accuracy, robustness, and stability, and nonparametric tests confirm the statistical significance of these improvements. The hybrid framework offers practical guidance for designing robust swarm optimizers and demonstrates substantial value for complex optimization problems.

Despite these strengths, the WDHOA incurs moderately higher computational cost than lightweight baselines and may lose diversity in very high-dimensional settings under static parameterization. Future research will explore adaptive parameter control, efficient implementations for large-scale scenes, and extensions to dynamic and multi-objective optimization, as well as applications to real-time engineering tasks. Beyond performance improvements, this study highlights how complementary biomimetic strategies can be designed to yield resilient population-based search.

## Figures and Tables

**Table 1:** Conceptual differences between the WDHOA and previously enhanced HOA variants.

Algorithms	Main Improvement Strategies	Type of Mechanism
**CDHOA**	Good point set initialization; Cauchy inverse CDF operator; differential mutation	Initialization; statistical perturbation
**MHOA**	Roulette wheel mechanism; Logistic–Tent chaotic perturbation factors; flexible position update	Chaotic mapping; perturbation
**AEDHOA**	Hierarchical random initialization; leader coordination; adaptive perturbation; dynamic search	Leader-based adaptive control
**WDHOA**	Dynamic grouping; weighted hybrid search; angle-based direction generation	New synergistic exploration and exploitation framework

**Table 2:** Summary of the CEC 2017 benchmark.

No.	Functions	F(x)
**Unimodal Functions**		
1	Shifted and Rotated Bent Cigar Function	100
3	Shifted and Rotated Zakharov Function	300
**Simple Multimodal Functions**		
4	Shifted and Rotated Rosenbrock’s Function	400
5	Shifted and Rotated Rastrigin’s Function	500
6	Shifted and Rotated Expanded Scaffer’s F6 Function	600
7	Shifted and Rotated Lunacek Bi-Rastrigin Function	700
8	Shifted and Rotated Non-Continuous Rastrigin’s Function	800
9	Shifted and Rotated Levy Function	900
10	Shifted and Rotated Schwefel’s Function	1000
**Hybrid Functions**		
11	Hybrid Function 1 (N = 3)	1100
12	Hybrid Function 2 (N = 3)	1200
13	Hybrid Function 3 (N = 3)	1300
14	Hybrid Function 4 (N = 4)	1400
15	Hybrid Function 5 (N = 4)	1500
16	Hybrid Function 6 (N = 4)	1600
17	Hybrid Function 6 (N = 5)	1700
18	Hybrid Function 6 (N = 5)	1800
19	Hybrid Function 6 (N = 5)	1900
20	Hybrid Function 6 (N = 6)	2000
**Composition Functions**		
21	Composition Function 1 (N = 3)	2100
22	Composition Function 2 (N = 3)	2200
23	Composition Function 3 (N = 4)	2300
24	Composition Function 4 (N = 4)	2400
25	Composition Function 5 (N = 5)	2500
26	Composition Function 6 (N = 5)	2600
27	Composition Function 7 (N = 6)	2700
28	Composition Function 8 (N = 6)	2800
29	Composition Function 9 (N = 3)	2900
30	Composition Function 10 (N = 3)	3000
Search Range: ${\left[-100,\;100\right]}^{D}$.		

**Table 3:** Summary of the CEC 2022 benchmark.

**No.**	**Functions**	F(x*)
**Unimodal Functions**		
1	Shifted and Rotated Bent Cigar Function	300
**Basic Functions**		
2	Shifted and full Rotated Rosenbrock’s Function	400
3	Shifted and full Rotated Expanded Scaffer’s F6 Function	600
4	Shifted and full Rotated Non-Continuous Rastrigin’s Function	800
5	Shifted and full Rotated Levy Function	900
**Hybrid Functions**		
6	Hybrid Function 1 (N = 3)	1800
7	Hybrid Function 2 (N = 6)	2000
8	Hybrid Function 3 (N = 5)	2200
**Composition Functions**		
9	Composition Function 1 (N = 5)	2300
10	Composition Function 2 (N = 4)	2400
11	Composition Function 3 (N = 5)	2600
12	Composition Function 4 (N = 6)	2700
Search Range: ${\left[-100,\;100\right]}^{D}$.		

**Table 4:** Parameter settings for all comparison algorithms.

**Algorithms**	**Parameter Name**	**Parameter Value**
WDHOA	θ, α	[0, 50°], [1,3]
HOA	θ, α	[0, 50°], [1,3]
LSHADE	$p,\;r^{arc},\;r^{N^{init}},\;H$	0.11, 2.6, 18, 6
SFOA	C	0.8
STOA	b	1
SCA	α	5
RIME	w	5
KOA	$T,\;\mu_{0},\;\gamma$	3, 0.1, 15
CPSOGSA	φ	2.05

**Table 5:** Results for 20 dimensions on the CEC 2022 benchmark.

Func.	Metric	WDHOA	HOA	LSHADE	SFOA	STOA	SCA	RIME	KOA	CPSOGSA
**F1**	Std.	3.2 × 10^7^	8.10 × 10^3^	0.627	2.21 × 10^4^	3.25 × 10^3^	3.69 × 10^3^	2.68 × 10^1^	8.04 × 10^3^	1.79 × 10^4^
**Avg.**	3.00 × 10^2^	2.83 × 10^4^	3.00 × 10^2^	8.72 × 10^4^	1.22 × 10^4^	1.46 × 10^4^	3.43 × 10^2^	4.89 × 10^4^	4.39 × 10^4^	
**Rank**	1	6	2	9	4	5	3	8	7	
**F2**	Std.	2.19 × 10^1^	3.99 × 10^2^	1.96 × 10^1^	1.00 × 10^3^	6.22 × 10^1^	8.85 × 10^1^	2.07 × 10^1^	5.43 × 10^2^	8.44 × 10^1^
**Avg.**	4.34 × 10^2^	1.47 × 10^3^	4.46 × 10^2^	1.97 × 10^3^	5.71 × 10^2^	7.55 × 10^2^	4.55 × 10^2^	2.60 × 10^3^	5.35 × 10^2^	
**Rank**	1	7	2	8	5	6	3	9	4	
**F3**	Std.	9.37 × 10^3^	9.8	0.334	1.35 × 10^1^	9.07	5.4	2.02	4.77	1.15 × 10^1^
**Avg.**	6.00 × 10^2^	6.57 × 10^2^	6.00 × 10^2^	6.85 × 10^2^	6.31 × 10^2^	6.45 × 10^2^	6.02 × 10^2^	6.81 × 10^2^	6.54 × 10^2^	
**Rank**	1	7	2	9	4	5	3	8	6	
**F4**	Std.	1.47 × 10^1^	1.72 × 10^1^	8.14	2.98 × 10^1^	2.27 × 10^1^	1.50 × 10^1^	2.08 × 10^1^	1.31 × 10^1^	3.12 × 10^1^
**Avg.**	8.68 × 10^2^	9.25 × 10^2^	8.31 × 10^2^	1.02 × 10^3^	8.89 × 10^2^	9.46 × 10^2^	8.57 × 10^2^	1.01 × 10^3^	9.33 × 10^2^	
**Rank**	3	5	1	9	4	7	2	8	6	
**F5**	Std.	4.60 × 10^1^	5.10 × 10^2^	1.29 × 10^2^	2.41 × 10^3^	3.36 × 10^2^	4.36 × 10^2^	1.42 × 10^2^	8.63 × 10^2^	1.02 × 10^3^
**Avg.**	9.12 × 10^2^	2.80 × 10^3^	9.69 × 10^2^	7.10 × 10^3^	1.96 × 10^3^	2.42 × 10^3^	1.02 × 10^3^	6.55 × 10^3^	3.95 × 10^3^	
**Rank**	1	6	2	9	4	5	3	8	7	
**F6**	Std.	3.50 × 10^1^	4.93 × 10^8^	8.04 × 10^1^	6.56 × 10^8^	1.31 × 10^6^	1.11 × 10^8^	6.86 × 10^3^	4.13 × 10^8^	2.70 × 10^5^
**Avg.**	1.86 × 10^3^	5.85 × 10^8^	1.96 × 10^3^	8.06 × 10^8^	6.74 × 10^5^	1.43 × 10^8^	1.11 × 10^4^	1.27 × 10^9^	5.97 × 10^4^	
**Rank**	1	7	2	8	5	6	3	9	4	
**F7**	Std.	5.56	5.29 × 10^1^	2.25 × 10^1^	7.78 × 10^1^	4.47 × 10^1^	2.36 × 10^1^	3.63 × 10^1^	3.86 × 10^1^	9.84 × 10^1^
**Avg.**	2.04 × 10^3^	2.19 × 10^3^	2.03 × 10^3^	2.30 × 10^3^	2.13 × 10^3^	2.15 × 10^3^	2.07 × 10^3^	2.21 × 10^3^	2.24 × 10^3^	
**Rank**	2	6	1	9	4	5	3	7	8	
**F8**	Std.	1.14	9.38 × 10^1^	4.05 × 10^1^	1.92 × 10^2^	6.81 × 10^1^	1.60 × 10^1^	3.77 × 10^1^	1.11 × 10^2^	1.33 × 10^2^
**Avg.**	2.23 × 10^3^	2.31 × 10^3^	2.24 × 10^3^	2.52 × 10^3^	2.33 × 10^3^	2.27 × 10^3^	2.24 × 10^3^	2.45 × 10^3^	2.41 × 10^3^	
**Rank**	1	5	2	9	6	4	3	8	7	
**F9**	Std.	2.80 × 10^13^	1.95 × 10^2^	1.15 × 10^−9^	2.56 × 10^2^	4.71 × 10^1^	3.56 × 10^1^	0.214	9.73 × 10^1^	4.08 × 10^1^
**Avg.**	2.48 × 10^3^	3.08 × 10^3^	2.48 × 10^3^	2.82 × 10^3^	2.56 × 10^3^	2.60 × 10^3^	2.48 × 10^3^	2.88 × 10^3^	2.52 × 10^3^	
**Rank**	1	9	2	7	5	6	3	8	4	
**F10**	Std.	1.46 × 10^2^	1.29 × 10^3^	5.57 × 10^1^	1.43 × 10^3^	1.06 × 10^3^	1.50 × 10^3^	2.26 × 10^2^	2.66 × 10^2^	9.83 × 10^2^
**Avg.**	2.55 × 10^3^	5.07 × 10^3^	2.54 × 10^3^	6.35 × 10^3^	5.04 × 10^3^	3.29 × 10^3^	2.75 × 10^3^	2.95 × 10^3^	4.56 × 10^3^	
**Rank**	2	8	1	9	7	5	3	4	6	
**F11**	Std.	2.54 × 10^1^	9.35 × 10^2^	1.30 × 10^−6^	6.54 × 10^4^	6.93 × 10^2^	6.68 × 10^2^	9.58	1.46 × 10^4^	1.50 × 10^4^
**Avg.**	2.91 × 10^3^	7.32 × 10^3^	2.90 × 10^3^	1.15 × 10^5^	5.39 × 10^3^	6.52 × 10^3^	2.95 × 10^3^	1.08 × 10^5^	1.17 × 10^4^	
**Rank**	2	6	1	9	4	5	3	8	7	
**F12**	Std.	5.85	1.69 × 10^2^	3.02 × 10^1^	6.75 × 10^1^	2.29 × 10^1^	3.28 × 10^1^	1.38 × 10^1^	7.49 × 10^1^	1.12 × 10^2^
**Avg.**	2.95 × 10^3^	3.79 × 10^3^	2.98 × 10^3^	3.11 × 10^3^	2.98 × 10^3^	3.07 × 10^3^	2.96 × 10^3^	3.48 × 10^3^	3.10 × 10^3^	
**Rank**	1	9	4	7	3	5	2	8	6	
**Average rank**	1.417	6.75	1.83	8.5	4.58	5.33	2.83	7.75	6	
Friedman Chi-square statistic = 82.755556										
Chi-square critical value (k = 9, N = 12, alpha = 0.05) = 15.507313 *p* -value = 1.361 × 10^−14^										

**Table 6:** Wilcoxon rank-sum test for 20 dimensions on the CEC 2022 benchmark.

Func.	HOA	LSHADE	SFOA	STOA	SCA	RIME	KOA	CPSOGSA
**F1**	3.02 × 10^−11^	3.02 × 10^−11^	3.02 × 10^−11^	3.02 × 10^−11^	3.02 × 10^−11^	3.02 × 10^−11^	3.02 × 10^−11^	3.02 × 10^−11^
**F2**	2.31 × 10^−11^	2.66 × 10^−5^	2.31 × 10^−11^	2.31 × 10^−11^	2.31 × 10^−11^	1.5 × 10^−6^	2.31 × 10^−11^	2.31 × 10^−11^
**F3**	3.02 × 10^−11^	5.6 × 10^−7^	3.02 × 10^−11^	3.02 × 10^−11^	3.02 × 10^−11^	3.02 × 10^−11^	3.02 × 10^−11^	3.02 × 10^−11^
**F4**	6.07 × 10^−11^	4.5 × 10^−11^	3.02 × 10^−11^	3.01 × 10^−4^	3.02 × 10^−11^	1.95 × 10^−3^	3.02 × 10^−11^	1.96 × 10^−10^
**F5**	3.02 × 10^−11^	4.8 × 10^−7^	3.02 × 10^−11^	3.02 × 10^−11^	3.02 × 10^−11^	3.49 × 10^−9^	3.02 × 10^−11^	3.02 × 10^−11^
**F6**	3.02 × 10^−11^	3.65 × 10^−8^	3.02 × 10^−11^	3.02 × 10^−11^	3.02 × 10^−11^	3.02 × 10^−11^	3.02 × 10^−11^	3.02 × 10^−11^
**F7**	3.02 × 10^−11^	8.15 × 10^−5^	3.02 × 10^−11^	3.02 × 10^−11^	3.02 × 10^−11^	3.18 × 10^−4^	3.02 × 10^−11^	3.02 × 10^−11^
**F8**	3.02 × 10^−11^	4.98 × 10^−4^	3.02 × 10^−11^	3.34 × 10^−11^	3.02 × 10^−11^	6.84 × 10^−1^	3.02 × 10^−11^	6.12 × 10^−10^
**F9**	1.25 × 10^−11^	1.09 × 10^−11^	1.25 × 10^−11^	1.25 × 10^−11^	1.25 × 10^−11^	1.25 × 10^−11^	1.25 × 10^−11^	1.25 × 10^−11^
**F10**	3.47 × 10^−10^	1.5 × 10^−2^	1.21 × 10^−10^	4.62 × 10^−10^	1.43 × 10^−5^	9.51 × 10^−6^	9.76 × 10^−10^	4.62 × 10^−10^
**F11**	1.72 × 10^−11^	5.55 × 10^−9^	1.72 × 10^−11^	1.72 × 10^−11^	1.72 × 10^−11^	5.55 × 10^−9^	1.72 × 10^−11^	1.72 × 10^−11^
**F12**	3.02 × 10^−11^	1.25 × 10^−7^	3.02 × 10^−11^	4.2 × 10^−10^	3.02 × 10^−11^	2.83 × 10^−8^	3.02 × 10^−11^	3.34 × 10^−11^

**Table 7:** Results for 10 dimensions on the CEC 2017 benchmark.

Func.	Metric	WDHOA	HOA	LSHADE	SFOA	STOA	SCA	RIME	KOA	CPSOGSA
**F1: 1**	**Std.**	0	1.98 × 10^9^	3.73 × 10^−15^	2.85 × 10^9^	2.22 × 10^8^	3.42 × 10^8^	5.51 × 10^3^	1.38 × 10^9^	2.46 × 10^8^
	**Avg.**	1 × 10^2^	4.85 × 10^9^	1 × 10^2^	3.51 × 10^9^	2.39 × 10^8^	9.43 × 10^8^	7.34 × 10^3^	4.67 × 10^9^	4.74 × 10^7^
**F3: 1**	**Std.**	0	2.64 × 10^3^	1.06 × 10^−14^	1.53 × 10^4^	1.78 × 10^3^	9.23 × 10^2^	1.34 × 10^−1^	4.82 × 10^3^	9.02 × 10^3^
	**Avg.**	3 × 10^2^	9.82 × 10^3^	3 × 10^2^	2.79 × 10^4^	2.12 × 10^3^	1.96 × 10^3^	3 × 10^2^	1.45 × 10^4^	7.2 × 10^3^
**F4: 1**	**Std.**	0	1.65 × 10^2^	0	1.26 × 10^2^	3.86 × 10^1^	1.4 × 10^1^	1.24 × 10^1^	1.11 × 10^2^	2.86 × 10^1^
	**Avg.**	4 × 10^2^	6.52 × 10^2^	4 × 10^2^	5.62 × 10^2^	4.51 × 10^2^	4.49 × 10^2^	4.08 × 10^2^	6.85 × 10^2^	4.22 × 10^2^
**F5: 2**	**Std.**	2.23 × 10^0^	1.65 × 10^1^	1.74 × 10^0^	1.97 × 10^1^	9.01 × 10^0^	8.15 × 10^0^	4.24 × 10^0^	9.09 × 10^0^	2.06 × 10^1^
	**Avg.**	5.06 × 10^2^	5.57 × 10^2^	5.05 × 10^2^	5.83 × 10^2^	5.3 × 10^2^	5.48 × 10^2^	5.13 × 10^2^	5.81 × 10^2^	5.5 × 10^2^
**F6: 2**	**Std.**	1.67 × 10^−4^	9.59 × 10^0^	2.75 × 10^−5^	1.37 × 10^1^	5.5 × 10^0^	4.33 × 10^0^	4.67 × 10^−2^	6.66 × 10^0^	1.74 × 10^1^
	**Avg.**	6 × 10^2^	6.28 × 10^2^	6 × 10^2^	6.53 × 10^2^	6.11 × 10^2^	6.19 × 10^2^	6 × 10^2^	6.45 × 10^2^	6.29 × 10^2^
**F7: 2**	**Std.**	3.76 × 10^0^	1.34 × 10^1^	1.69 × 10^0^	5.04 × 10^1^	1.77 × 10^1^	9 × 10^0^	7.45 × 10^0^	2.8 × 10^1^	3.61 × 10^1^
	**Avg.**	7.19 × 10^2^	7.64 × 10^2^	7.15 × 10^2^	8.32 × 10^2^	7.58 × 10^2^	7.79 × 10^2^	7.24 × 10^2^	9.07 × 10^2^	7.71 × 10^2^
**F8: 2**	**Std.**	2.28 × 10^0^	5.91 × 10^0^	1.64 × 10^0^	1.89 × 10^1^	7.42 × 10^0^	6.75 × 10^0^	5.76 × 10^0^	8.16 × 10^0^	1.6 × 10^1^
	**Avg.**	8.06 × 10^2^	8.34 × 10^2^	8.05 × 10^2^	8.8 × 10^2^	8.24 × 10^2^	8.41 × 10^2^	8.15 × 10^2^	8.78 × 10^2^	8.46 × 10^2^
**F9: 1**	**Std.**	3.6 × 10^−2^	1.37 × 10^2^	1.39 × 10^−1^	5.46 × 10^2^	5.88 × 10^1^	5.38 × 10^1^	1.98 × 10^−1^	2.82 × 10^2^	6.09 × 10^2^
	**Avg.**	9 × 10^2^	1.13 × 10^3^	9 × 10^2^	2.09 × 10^3^	9.84 × 10^2^	1.02 × 10^3^	9 × 10^2^	2.06 × 10^3^	1.86 × 10^3^
**F10: 2**	**Std.**	1.39 × 10^2^	2.73 × 10^2^	1.49 × 10^2^	2.05 × 10^2^	2.32 × 10^2^	2.68 × 10^2^	2.08 × 10^2^	1.75 × 10^2^	3.53 × 10^2^
	**Avg.**	1.4 × 10^3^	2.25 × 10^3^	1.3 × 10^3^	2.76 × 10^3^	1.81 × 10^3^	2.4 × 10^3^	1.48 × 10^3^	2.66 × 10^3^	2.27 × 10^3^
**F11: 1**	**Std.**	1.5 × 10^0^	1.35 × 10^3^	3.93 × 10^0^	1.01 × 10^3^	1.06 × 10^2^	3.92 × 10^1^	6.61 × 10^0^	2.06 × 10^2^	5.65 × 10^2^
	**Avg.**	1.1 × 10^3^	2.54 × 10^3^	1.1 × 10^3^	2.22 × 10^3^	1.27 × 10^3^	1.21 × 10^3^	1.11 × 10^3^	1.67 × 10^3^	1.32 × 10^3^
**F12: 1**	**Std.**	1.09 × 10^2^	5.67 × 10^6^	2.36 × 10^2^	1.51 × 10^8^	4.29 × 10^6^	1.63 × 10^7^	2.26 × 10^4^	6.3 × 10^7^	3.92 × 10^6^
	**Avg.**	1.37 × 10^3^	7.35 × 10^6^	1.66 × 10^3^	9.73 × 10^7^	3.68 × 10^6^	2.12 × 10^7^	2.27 × 10^4^	1.21 × 10^8^	1.03 × 10^6^
**F13: 1**	**Std.**	2.37 × 10^0^	9.56 × 10^6^	8.22 × 10^0^	1.52 × 10^7^	1.48 × 10^4^	3.81 × 10^4^	1.14 × 10^4^	6.91 × 10^5^	1.22 × 10^4^
	**Avg.**	1.31 × 10^3^	2.54 × 10^6^	1.31 × 10^3^	3.37 × 10^6^	1.9 × 10^4^	4.98 × 10^4^	1.16 × 10^4^	1.05 × 10^6^	1.67 × 10^4^
**F14: 1**	**Std.**	5.95 × 10^0^	3.12 × 10^3^	9.88 × 10^0^	2.81 × 10^4^	2 × 10^3^	7.88 × 10^2^	1.32 × 10^3^	7.98 × 10^2^	5.65 × 10^3^
	**Avg.**	1.41 × 10^3^	4.26 × 10^3^	1.41 × 10^3^	1.81 × 10^4^	2.46 × 10^3^	1.93 × 10^3^	2.28 × 10^3^	2.35 × 10^3^	6.24 × 10^3^
**F15: 1**	**Std.**	5.49 × 10^−1^	3.31 × 10^3^	4.01 × 10^0^	4.08 × 10^4^	2.33 × 10^3^	1.79 × 10^3^	5.41 × 10^3^	5.09 × 10^3^	1.07 × 10^4^
	**Avg.**	1.5 × 10^3^	7.82 × 10^3^	1.5 × 10^3^	3.57 × 10^4^	3.98 × 10^3^	3.14 × 10^3^	4.74 × 10^3^	9.44 × 10^3^	1.17 × 10^4^
**F16: 1**	**Std.**	2.44 × 10^0^	9.36 × 10^1^	6.3 × 10^1^	1.87 × 10^2^	1.37 × 10^2^	9.39 × 10^1^	1.25 × 10^2^	9.48 × 10^1^	2.17 × 10^2^
	**Avg.**	1.6 × 10^3^	2 × 10^3^	1.64 × 10^3^	2.08 × 10^3^	1.77 × 10^3^	1.77 × 10^3^	1.75 × 10^3^	2 × 10^3^	1.95 × 10^3^
**F17: 2**	**Std.**	5.44 × 10^0^	5.47 × 10^1^	9.57 × 10^0^	1.03 × 10^2^	4.06 × 10^1^	1.59 × 10^1^	4.71 × 10^1^	3.81 × 10^1^	1.07 × 10^2^
	**Avg.**	1.71 × 10^3^	1.8 × 10^3^	1.71 × 10^3^	1.91 × 10^3^	1.78 × 10^3^	1.79 × 10^3^	1.77 × 10^3^	1.88 × 10^3^	1.86 × 10^3^
**F18: 1**	**Std.**	6.94 × 10^0^	5.54 × 10^7^	2.18 × 10^1^	1.13 × 10^7^	1.76 × 10^4^	1.73 × 10^5^	9.34 × 10^3^	2.15 × 10^6^	1.26 × 10^4^
	**Avg.**	1.81 × 10^3^	1.88 × 10^7^	1.83 × 10^3^	4.66 × 10^6^	3.38 × 10^4^	1.99 × 10^5^	1.31 × 10^4^	2.27 × 10^6^	2.58 × 10^4^
**F19: 1**	**Std.**	4.33 × 10^−1^	8.12 × 10^4^	2.75 × 10^0^	1.03 × 10^5^	7.69 × 10^3^	5.37 × 10^3^	4.06 × 10^3^	1.56 × 10^4^	9.46 × 10^3^
	**Avg.**	1.9 × 10^3^	4.51 × 10^4^	1.9 × 10^3^	6.9 × 10^4^	8.77 × 10^3^	7.47 × 10^3^	4.96 × 10^3^	1.89 × 10^4^	9.64 × 10^3^
**F20: 1**	**Std.**	5.02 × 10^0^	6.88 × 10^1^	2.21 × 10^1^	9.78 × 10^1^	6.57 × 10^1^	3.66 × 10^1^	5.05 × 10^1^	3.47 × 10^1^	1.2 × 10^2^
	**Avg.**	2 × 10^3^	2.15 × 10^3^	2 × 10^3^	2.26 × 10^3^	2.11 × 10^3^	2.11 × 10^3^	2.04 × 10^3^	2.17 × 10^3^	2.21 × 10^3^
**F21: 2**	**Std.**	4.72 × 10^1^	3.79 × 10^1^	3.69 × 10^1^	1.41 × 10^1^	2.29 × 10^0^	6.77 × 10^1^	4.14 × 10^1^	2.15 × 10^1^	4.91 × 10^1^
	**Avg.**	2.23 × 10^3^	2.34 × 10^3^	2.29 × 10^3^	2.27 × 10^3^	2.2 × 10^3^	2.27 × 10^3^	2.3 × 10^3^	2.27 × 10^3^	2.34 × 10^3^
**F22: 1**	**Std.**	2.25 × 10^1^	1.24 × 10^2^	1.42 × 10^1^	5.32 × 10^2^	6.59 × 10^2^	3.44 × 10^1^	1.61 × 10^0^	1.42 × 10^2^	4.21 × 10^2^
	**Avg.**	2.29 × 10^3^	2.52 × 10^3^	2.3 × 10^3^	2.7 × 10^3^	3.04 × 10^3^	2.37 × 10^3^	2.3 × 10^3^	2.61 × 10^3^	2.45 × 10^3^
**F23: 2**	**Std.**	2.52 × 10^0^	3.14 × 10^1^	3.17 × 10^0^	1.61 × 10^1^	1.04 × 10^1^	9.32 × 10^0^	7.12 × 10^0^	1.59 × 10^1^	2.72 × 10^1^
	**Avg.**	2.61 × 10^3^	2.74 × 10^3^	2.61 × 10^3^	2.67 × 10^3^	2.63 × 10^3^	2.66 × 10^3^	2.62 × 10^3^	2.7 × 10^3^	2.66 × 10^3^
**F24: 1**	**Std.**	1.01 × 10^2^	1.02 × 10^2^	4.32 × 10^1^	1.4 × 10^1^	1.06 × 10^1^	6.89 × 10^1^	6.5 × 10^1^	5.75 × 10^1^	6.42 × 10^1^
	**Avg.**	2.7 × 10^3^	2.81 × 10^3^	2.73 × 10^3^	2.81 × 10^3^	2.76 × 10^3^	2.76 × 10^3^	2.74 × 10^3^	2.75 × 10^3^	2.79 × 10^3^
**F25: 1**	**Std.**	2.33 × 10^1^	1.29 × 10^2^	2.3 × 10^1^	1.47 × 10^2^	2.33 × 10^1^	2.01 × 10^1^	2.41 × 10^1^	6.84 × 10^1^	7.15 × 10^1^
	**Avg.**	2.92 × 10^3^	3.1 × 10^3^	2.92 × 10^3^	3.12 × 10^3^	2.94 × 10^3^	2.96 × 10^3^	2.93 × 10^3^	3.18 × 10^3^	2.94 × 10^3^
**F26: 1**	**Std.**	3.33 × 10^1^	3.46 × 10^2^	1.66 × 10^2^	5.63 × 10^2^	4.99 × 10^2^	4.46 × 10^1^	2.13 × 10^2^	1.44 × 10^2^	5.17 × 10^2^
	**Avg.**	2.91 × 10^3^	3.61 × 10^3^	2.96 × 10^3^	3.7 × 10^3^	3.52 × 10^3^	3.11 × 10^3^	2.95 × 10^3^	3.52 × 10^3^	3.48 × 10^3^
**F27: 1**	**Std.**	1.07 × 10^0^	3.26 × 10^1^	5.25 × 10^0^	9.87 × 10^0^	2.58 × 10^0^	3.11 × 10^0^	7.44 × 10^0^	1.52 × 10^1^	4.01 × 10^1^
	**Avg.**	3.09 × 10^3^	3.24 × 10^3^	3.09 × 10^3^	3.11 × 10^3^	3.09 × 10^3^	3.1 × 10^3^	3.1 × 10^3^	3.17 × 10^3^	3.15 × 10^3^
**F28: 1**	**Std.**	1.27 × 10^2^	1.26 × 10^2^	1.26 × 10^2^	1.21 × 10^2^	1.04 × 10^2^	7.21 × 10^1^	1.54 × 10^2^	7.47 × 10^1^	1.06 × 10^2^
	**Avg.**	3.19 × 10^3^	3.69 × 10^3^	3.34 × 10^3^	3.43 × 10^3^	3.32 × 10^3^	3.3 × 10^3^	3.3 × 10^3^	3.46 × 10^3^	3.35 × 10^3^
**F29: 2**	**Std.**	1.54 × 10^1^	7.02 × 10^1^	2.24 × 10^1^	1.03 × 10^2^	4.78 × 10^1^	4.35 × 10^1^	4.23 × 10^1^	4.62 × 10^1^	9.7 × 10^1^
	**Avg.**	3.15 × 10^3^	3.3 × 10^3^	3.15 × 10^3^	3.41 × 10^3^	3.19 × 10^3^	3.25 × 10^3^	3.19 × 10^3^	3.36 × 10^3^	3.32 × 10^3^
**F30: 1**	**Std.**	2.82 × 10^5^	4.91 × 10^6^	5.14 × 10^5^	1.11 × 10^5^	2.51 × 10^5^	9.99 × 10^5^	4.59 × 10^5^	3.42 × 10^6^	1.21 × 10^6^
	**Avg.**	1.15 × 10^5^	3.45 × 10^6^	3.75 × 10^5^	8.65 × 10^5^	2.4 × 10^5^	1.27 × 10^6^	2.63 × 10^5^	7.8 × 10^6^	8.24 × 10^5^
**Average rank**		1.31 × 10^0^	7.24 × 10^0^	2.07 × 10^0^	8.14 × 10^0^	4.48 × 10^0^	5.1 × 10^0^	3.21 × 10^0^	7.41 × 10^0^	6 × 10^0^
Friedman Chi-square statistic = 182.343399										
Chi-square critical value (k = 9, N = 29, alpha = 0.05) = 15.507313 *p*-value = 3.315 × 10^−35^										

**Table 8:** Results for 30 dimensions on the CEC 2017 benchmark.

Func.	Metric	WDHOA	HOA	LSHADE	SFOA	STOA	SCA	RIME	KOA	CPSOGSA
**F1: 1**	**Std.**	5.33 × 10^1^	7.92 × 10^9^	9.27 × 10^4^	1.6 × 10^10^	4.03 × 10^9^	3.94 × 10^9^	2.11 × 10^5^	6.11 × 10^9^	3.42 × 10^9^
	**Avg.**	1.25 × 10^2^	3.85 × 10^10^	2.09 × 10^4^	4.21 × 10^10^	9.95 × 10^9^	1.79 × 10^10^	6.25 × 10^5^	6.72 × 10^10^	1.89 × 10^9^
**F3: 1**	**Std.**	2.79 × 10^1^	6.4 × 10^3^	1.59 × 10^3^	8.7 × 10^4^	1.02 × 10^4^	1.28 × 10^4^	6.74 × 10^3^	2.03 × 10^4^	5.53 × 10^4^
	**Avg.**	3.27 × 10^2^	7.37 × 10^4^	2.35 × 10^3^	2.4 × 10^5^	5.54 × 10^4^	6.7 × 10^4^	1.35 × 10^4^	1.57 × 10^5^	2 × 10^5^
**F4: 1**	**Std.**	3.35 × 10^1^	1.9 × 10^3^	2.64 × 10^1^	7.31 × 10^3^	1.75 × 10^2^	6.06 × 10^2^	2.32 × 10^1^	2.99 × 10^3^	1.87 × 10^2^
	**Avg.**	4.4 × 10^2^	7.4 × 10^3^	4.99 × 10^2^	1.26 × 10^4^	8.77 × 10^2^	2.61 × 10^3^	5.08 × 10^2^	1.58 × 10^4^	6.73 × 10^2^
**F5: 3**	**Std.**	2.11 × 10^1^	3.21 × 10^1^	2.06 × 10^1^	5.75 × 10^1^	2.89 × 10^1^	2.54 × 10^1^	3.71 × 10^1^	2.51 × 10^1^	6.13 × 10^1^
	**Avg.**	6.18 × 10^2^	8.15 × 10^2^	5.8 × 10^2^	9.73 × 10^2^	7.09 × 10^2^	8.15 × 10^2^	6.04 × 10^2^	9.97 × 10^2^	7.83 × 10^2^
**F6: 1**	**Std.**	9.23 × 10^−2^	7.4 × 10^0^	1.03 × 10^0^	1.81 × 10^1^	8.25 × 10^0^	7.02 × 10^0^	4.16 × 10^0^	6.73 × 10^0^	1.05 × 10^1^
	**Avg.**	6 × 10^2^	6.68 × 10^2^	6.02 × 10^2^	7.05 × 10^2^	6.4 × 10^2^	6.61 × 10^2^	6.08 × 10^2^	6.97 × 10^2^	6.67 × 10^2^
**F7: 3**	**Std.**	2.21 × 10^1^	7.33 × 10^1^	2.74 × 10^1^	4.09 × 10^2^	5.45 × 10^1^	4.58 × 10^1^	2.44 × 10^1^	1.63 × 10^2^	1.86 × 10^2^
	**Avg.**	8.74 × 10^2^	1.24 × 10^3^	8.54 × 10^2^	1.82 × 10^3^	1.11 × 10^3^	1.21 × 10^3^	8.46 × 10^2^	2.48 × 10^3^	1.65 × 10^3^
**F8: 3**	**Std.**	2.06 × 10^1^	2.58 × 10^1^	1.84 × 10^1^	6.9 × 10^1^	3.15 × 10^1^	2.03 × 10^1^	2.38 × 10^1^	1.95 × 10^1^	5.83 × 10^1^
	**Avg.**	9.1 × 10^2^	1.05 × 10^3^	8.75 × 10^2^	1.23 × 10^3^	9.91 × 10^2^	1.09 × 10^3^	8.97 × 10^2^	1.24 × 10^3^	1.05 × 10^3^
**F9: 1**	**Std.**	2.02 × 10^2^	1.26 × 10^3^	3.44 × 10^2^	7.4 × 10^3^	1.97 × 10^3^	1.67 × 10^3^	1.15 × 10^3^	1.79 × 10^3^	2.53 × 10^3^
	**Avg.**	9.73 × 10^2^	7.03 × 10^3^	1.43 × 10^3^	2.12 × 10^4^	6.13 × 10^3^	7.41 × 10^3^	2.38 × 10^3^	2.01 × 10^4^	9.18 × 10^3^
**F10: 4**	**Std.**	5.57 × 10^2^	6.22 × 10^2^	5.83 × 10^2^	2.44 × 10^2^	7.4 × 10^2^	3.52 × 10^2^	5.48 × 10^2^	3.26 × 10^2^	5.22 × 10^2^
	**Avg.**	6.59 × 10^3^	7.39 × 10^3^	4.93 × 10^3^	9.1 × 10^3^	6.77 × 10^3^	8.66 × 10^3^	4.59 × 10^3^	8.98 × 10^3^	5.33 × 10^3^
**F11: 1**	**Std.**	2.99 × 10^1^	1.95 × 10^3^	4.62 × 10^1^	7.92 × 10^3^	1.08 × 10^3^	1.09 × 10^3^	5.34 × 10^1^	2.16 × 10^3^	4.43 × 10^3^
	**Avg.**	1.16 × 10^3^	6.79 × 10^3^	1.23 × 10^3^	1.85 × 10^4^	2.58 × 10^3^	3.44 × 10^3^	1.29 × 10^3^	1.1 × 10^4^	3.44 × 10^3^
**F12: 1**	**Std.**	2.58 × 10^4^	2.05 × 10^9^	3.34 × 10^5^	3.78 × 10^9^	3.49 × 10^8^	5.37 × 10^8^	8.45 × 10^6^	1.62 × 10^9^	4.58 × 10^8^
	**Avg.**	3.31 × 10^4^	6.51 × 10^9^	1.2 × 10^5^	5.83 × 10^9^	4.02 × 10^8^	2.18 × 10^9^	1.18 × 10^7^	9.39 × 10^9^	1.51 × 10^8^
**F13: 1**	**Std.**	1.54 × 10^4^	1.52 × 10^9^	1.33 × 10^4^	3.57 × 10^9^	1.28 × 10^8^	4.05 × 10^8^	1.52 × 10^5^	1.83 × 10^9^	8.04 × 10^5^
	**Avg.**	6.06 × 10^3^	2.26 × 10^9^	1.39 × 10^4^	3.46 × 10^9^	1.22 × 10^8^	8.38 × 10^8^	9.33 × 10^4^	4.86 × 10^9^	2.01 × 10^5^
**F14: 1**	**Std.**	1.22 × 10^1^	8.31 × 10^5^	8.47 × 10^1^	3.69 × 10^6^	8.37 × 10^5^	6.28 × 10^5^	3.91 × 10^4^	1.27 × 10^6^	4.37 × 10^5^
	**Avg.**	1.49 × 10^3^	1.44 × 10^6^	1.62 × 10^3^	3.73 × 10^6^	4.58 × 10^5^	6.18 × 10^5^	4.73 × 10^4^	2.41 × 10^6^	3.81 × 10^5^
**F15: 1**	**Std.**	3.35 × 10^1^	1.05 × 10^8^	4.73 × 10^2^	3.91 × 10^8^	1.72 × 10^7^	3.76 × 10^7^	9.44 × 10^3^	2.71 × 10^8^	2.97 × 10^4^
	**Avg.**	1.6 × 10^3^	9.06 × 10^7^	1.96 × 10^3^	3.25 × 10^8^	1.2 × 10^7^	3.51 × 10^7^	1.02 × 10^4^	6.74 × 10^8^	2.61 × 10^4^
**F16: 2**	**Std.**	2.16 × 10^2^	6.6 × 10^2^	2.26 × 10^2^	5.94 × 10^2^	3.68 × 10^2^	2.3 × 10^2^	3.08 × 10^2^	3.56 × 10^2^	3.63 × 10^2^
	**Avg.**	2.37 × 10^3^	4.44 × 10^3^	2.26 × 10^3^	4.95 × 10^3^	3.23 × 10^3^	4 × 10^3^	2.66 × 10^3^	5.23 × 10^3^	3.16 × 10^3^
**F17: 1**	**Std.**	6.9 × 10^1^	3.97 × 10^2^	9.42 × 10^1^	4.29 × 10^2^	1.93 × 10^2^	1.85 × 10^2^	2.38 × 10^2^	2.33 × 10^2^	3.16 × 10^2^
	**Avg.**	1.87 × 10^3^	2.99 × 10^3^	1.88 × 10^3^	3.4 × 10^3^	2.4 × 10^3^	2.71 × 10^3^	2.18 × 10^3^	3.53 × 10^3^	2.61 × 10^3^
**F18: 1**	**Std.**	2.75 × 10^1^	2.23 × 10^7^	2.41 × 10^4^	6.1 × 10^7^	2.55 × 10^6^	6.31 × 10^6^	8.57 × 10^5^	2.1 × 10^7^	2.87 × 10^6^
	**Avg.**	1.91 × 10^3^	1.69 × 10^7^	2.7 × 10^4^	5.54 × 10^7^	2.03 × 10^6^	9.77 × 10^6^	1.19 × 10^6^	3.9 × 10^7^	2.56 × 10^6^
**F19: 1**	**Std.**	7.07 × 10^0^	6.07 × 10^7^	8.1 × 10^1^	7.28 × 10^8^	1.31 × 10^7^	2.93 × 10^7^	1.08 × 10^4^	3.46 × 10^8^	1.91 × 10^5^
	**Avg.**	1.94 × 10^3^	5.4 × 10^7^	2.08 × 10^3^	5.09 × 10^8^	1.21 × 10^7^	6.13 × 10^7^	1.08 × 10^4^	9.47 × 10^8^	5.33 × 10^4^
**F20: 2**	**Std.**	9.81 × 10^1^	1.85 × 10^2^	1.03 × 10^2^	1.94 × 10^2^	1.72 × 10^2^	1.18 × 10^2^	1.93 × 10^2^	8 × 10^1^	2.62 × 10^2^
	**Avg.**	2.22 × 10^3^	2.71 × 10^3^	2.18 × 10^3^	3.31 × 10^3^	2.68 × 10^3^	2.9 × 10^3^	2.47 × 10^3^	3.1 × 10^3^	2.91 × 10^3^
**F21: 3**	**Std.**	2.94 × 10^1^	3.71 × 10^1^	1.65 × 10^1^	8.79 × 10^1^	2.82 × 10^1^	2.28 × 10^1^	2.98 × 10^1^	2.85 × 10^1^	5.51 × 10^1^
	**Avg.**	2.42 × 10^3^	2.6 × 10^3^	2.38 × 10^3^	2.75 × 10^3^	2.5 × 10^3^	2.6 × 10^3^	2.42 × 10^3^	2.74 × 10^3^	2.58 × 10^3^
**F22: 3**	**Std.**	2.83 × 10^3^	1.51 × 10^3^	1.24 × 10^3^	1.61 × 10^3^	1.28 × 10^3^	1.84 × 10^3^	2.09 × 10^3^	7.91 × 10^2^	1.94 × 10^3^
	**Avg.**	5.39 × 10^3^	7.83 × 10^3^	2.72 × 10^3^	1.01 × 10^4^	8.25 × 10^3^	9.41 × 10^3^	4.94 × 10^3^	9.18 × 10^3^	6.55 × 10^3^
**F23: 1**	**Std.**	2.17 × 10^1^	1.64 × 10^2^	4.34 × 10^1^	1.02 × 10^2^	4.25 × 10^1^	4.33 × 10^1^	3.36 × 10^1^	7.09 × 10^1^	9.77 × 10^1^
	**Avg.**	2.76 × 10^3^	3.51 × 10^3^	2.79 × 10^3^	3.2 × 10^3^	2.86 × 10^3^	3.06 × 10^3^	2.77 × 10^3^	3.39 × 10^3^	3.09 × 10^3^
**F24: 2**	**Std.**	1.01 × 10^2^	1.02 × 10^2^	4.32 × 10^1^	1.4 × 10^1^	1.06 × 10^1^	6.89 × 10^1^	6.5 × 10^1^	5.75 × 10^1^	6.42 × 10^1^
	**Avg.**	2.7 × 10^3^	2.81 × 10^3^	2.73 × 10^3^	2.81 × 10^3^	2.76 × 10^3^	2.76 × 10^3^	2.74 × 10^3^	2.75 × 10^3^	2.79 × 10^3^
**F25: 1**	**Std.**	2.33 × 10^1^	1.29 × 10^2^	2.3 × 10^1^	1.47 × 10^2^	2.33 × 10^1^	2.01 × 10^1^	2.41 × 10^1^	6.84 × 10^1^	7.15 × 10^1^
	**Avg.**	2.92 × 10^3^	3.1 × 10^3^	2.92 × 10^3^	3.12 × 10^3^	2.94 × 10^3^	2.96 × 10^3^	2.93 × 10^3^	3.18 × 10^3^	2.94 × 10^3^
**F26: 1**	**Std.**	3.33 × 10^1^	3.46 × 10^2^	1.66 × 10^2^	5.63 × 10^2^	4.99 × 10^2^	4.46 × 10^1^	2.13 × 10^2^	1.44 × 10^2^	5.17 × 10^2^
	**Avg.**	2.91 × 10^3^	3.61 × 10^3^	2.96 × 10^3^	3.7 × 10^3^	3.52 × 10^3^	3.11 × 10^3^	2.95 × 10^3^	3.52 × 10^3^	3.48 × 10^3^
**F27: 1**	**Std.**	1.07 × 10^0^	3.26 × 10^1^	5.25 × 10^0^	9.87 × 10^0^	2.58 × 10^0^	3.11 × 10^0^	7.44 × 10^0^	1.52 × 10^1^	4.01 × 10^1^
	**Avg.**	3.09 × 10^3^	3.24 × 10^3^	3.09 × 10^3^	3.11 × 10^3^	3.09 × 10^3^	3.1 × 10^3^	3.1 × 10^3^	3.17 × 10^3^	3.15 × 10^3^
**F28: 1**	**Std.**	1.27 × 10^2^	1.26 × 10^2^	1.26 × 10^2^	1.21 × 10^2^	1.04 × 10^2^	7.21 × 10^1^	1.54 × 10^2^	7.47 × 10^1^	1.06 × 10^2^
	**Avg.**	3.19 × 10^3^	3.69 × 10^3^	3.34 × 10^3^	3.43 × 10^3^	3.32 × 10^3^	3.3 × 10^3^	3.3 × 10^3^	3.46 × 10^3^	3.35 × 10^3^
**F29: 2**	**Std.**	1.54 × 10^1^	7.02 × 10^1^	2.24 × 10^1^	1.03 × 10^2^	4.78 × 10^1^	4.35 × 10^1^	4.23 × 10^1^	4.62 × 10^1^	9.7 × 10^1^
	**Avg.**	3.15 × 10^3^	3.3 × 10^3^	3.15 × 10^3^	3.41 × 10^3^	3.19 × 10^3^	3.25 × 10^3^	3.19 × 10^3^	3.36 × 10^3^	3.32 × 10^3^
**F30: 1**	**Std.**	2.82 × 10^5^	4.91 × 10^6^	5.14 × 10^5^	1.11 × 10^5^	2.51 × 10^5^	9.99 × 10^5^	4.59 × 10^5^	3.42 × 10^6^	1.21 × 10^6^
	**Avg.**	1.15 × 10^5^	3.45 × 10^6^	3.75 × 10^5^	8.65 × 10^5^	2.4 × 10^5^	1.27 × 10^6^	2.63 × 10^5^	7.8 × 10^6^	8.24 × 10^5^
**Average rank**		1.59 × 10^0^	6.93 × 10^0^	1.86 × 10^0^	8.14 × 10^0^	4.52 × 10^0^	5.97 × 10^0^	2.59 × 10^0^	8.48 × 10^0^	4.93 × 10^0^
Friedman Chi-square statistic = 209.581609										
Chi-square critical value (k = 9, N = 29, alpha = 0.05) = 15.507313 *p*-value = 6.099 × 10^−41^										

**Table 9:** Results for 50 dimensions on the CEC 2017 benchmark.

Func.	Metric	WDHOA	HOA	LSHADE	SFOA	STOA	SCA	RIME	KOA	CPSOGSA
**F1: 1**	**Std.**	6.66 × 10^3^	8.53 × 10^9^	3.23 × 10^8^	4.08 × 10^10^	9.58 × 10^9^	8.46 × 10^9^	2.13 × 10^6^	1.54 × 10^10^	9.07 × 10^9^
	**Avg.**	5.39 × 10^3^	8.52 × 10^10^	1.74 × 10^8^	1.21 × 10^11^	3.76 × 10^10^	6.09 × 10^10^	7.06 × 10^6^	1.66 × 10^11^	6.53 × 10^9^
**F3: 1**	**Std.**	6.46 × 10^3^	1.35 × 10^4^	1.09 × 10^4^	1.56 × 10^5^	2.4 × 10^4^	3.07 × 10^4^	3.23 × 10^4^	4.61 × 10^4^	1.41 × 10^5^
	**Avg.**	2.12 × 10^4^	1.52 × 10^5^	3.38 × 10^4^	5.84 × 10^5^	1.35 × 10^5^	1.82 × 10^5^	1.29 × 10^5^	3.31 × 10^5^	4.46 × 10^5^
**F4: 1**	**Std.**	5.18 × 10^1^	3.9 × 10^3^	6.63 × 10^1^	1.27 × 10^4^	9.15 × 10^2^	2.44 × 10^3^	8.18 × 10^1^	6.07 × 10^3^	8.62 × 10^2^
	**Avg.**	5.3 × 10^2^	2.29 × 10^4^	6.36 × 10^2^	3.71 × 10^4^	3.81 × 10^3^	1.13 × 10^4^	6.58 × 10^2^	5.19 × 10^4^	1.73 × 10^3^
**F5: 3**	**Std.**	4.3 × 10^1^	4.34 × 10^1^	4.04 × 10^1^	8.84 × 10^1^	4.85 × 10^1^	3.7 × 10^1^	4.12 × 10^1^	3.11 × 10^1^	8.47 × 10^1^
	**Avg.**	8.2 × 10^2^	1.07 × 10^3^	7.3 × 10^2^	1.34 × 10^3^	9.16 × 10^2^	1.12 × 10^3^	7.32 × 10^2^	1.44 × 10^3^	1.08 × 10^3^
**F6: 1**	**Std.**	5.87 × 10^−1^	5.88 × 10^0^	4.64 × 10^0^	1.97 × 10^1^	8.51 × 10^0^	5.99 × 10^0^	9.18 × 10^0^	6.24 × 10^0^	8.56 × 10^0^
	**Avg.**	6.01 × 10^2^	6.83 × 10^2^	6.17 × 10^2^	7.23 × 10^2^	6.65 × 10^2^	6.81 × 10^2^	6.23 × 10^2^	7.2 × 10^2^	6.77 × 10^2^
**F7: 2**	**Std.**	4.63 × 10^1^	9.99 × 10^1^	1.1 × 10^2^	8.47 × 10^2^	7.76 × 10^1^	1.04 × 10^2^	5.5 × 10^1^	2.2 × 10^2^	3.53 × 10^2^
	**Avg.**	1.13 × 10^3^	1.81 × 10^3^	1.23 × 10^3^	3.22 × 10^3^	1.61 × 10^3^	1.82 × 10^3^	1.04 × 10^3^	4.58 × 10^3^	3.02 × 10^3^
**F8: 3**	**Std.**	3.4 × 10^1^	3.66 × 10^1^	3.45 × 10^1^	1.39 × 10^2^	4.82 × 10^1^	2.75 × 10^1^	5.47 × 10^1^	4.03 × 10^1^	8.11 × 10^1^
	**Avg.**	1.12 × 10^3^	1.41 × 10^3^	1.02 × 10^3^	1.68 × 10^3^	1.25 × 10^3^	1.43 × 10^3^	1.02 × 10^3^	1.73 × 10^3^	1.37 × 10^3^
**F9: 1**	**Std.**	1.4 × 10^3^	4.3 × 10^3^	2.64 × 10^3^	1.73 × 10^4^	3.83 × 10^3^	4.5 × 10^3^	4.78 × 10^3^	4.62 × 10^3^	4.59 × 10^3^
	**Avg.**	2.83 × 10^3^	2.63 × 10^4^	8.25 × 10^3^	6.52 × 10^4^	2 × 10^4^	3.09 × 10^4^	9.58 × 10^3^	6.16 × 10^4^	2.28 × 10^4^
**F10: 4**	**Std.**	7.23 × 10^2^	7.82 × 10^2^	6.5 × 10^2^	4.52 × 10^2^	1.06 × 10^3^	4.99 × 10^2^	7.79 × 10^2^	4.08 × 10^2^	1.1 × 10^3^
	**Avg.**	1.22 × 10^4^	1.31 × 10^4^	9.25 × 10^3^	1.59 × 10^4^	1.28 × 10^4^	1.52 × 10^4^	7.74 × 10^3^	1.54 × 10^4^	8.55 × 10^3^
**F11: 1**	**Std.**	5.21 × 10^1^	2.96 × 10^3^	9.39 × 10^1^	2.67 × 10^4^	3.12 × 10^3^	2.99 × 10^3^	9.56 × 10^1^	5.01 × 10^3^	6.62 × 10^3^
	**Avg.**	1.33 × 10^3^	1.95 × 10^4^	1.45 × 10^3^	5.12 × 10^4^	7.58 × 10^3^	1.03 × 10^4^	1.56 × 10^3^	3.49 × 10^4^	1.19 × 10^4^
**F12: 1**	**Std.**	3.53 × 10^6^	9.63 × 10^9^	3.18 × 10^6^	2 × 10^10^	2.98 × 10^9^	3.45 × 10^9^	4.74 × 10^7^	8.99 × 10^9^	3.34 × 10^9^
	**Avg.**	1.67 × 10^6^	5.29 × 10^10^	4.35 × 10^6^	4.61 × 10^10^	6.36 × 10^9^	1.8 × 10^10^	9.82 × 10^7^	6.42 × 10^10^	1.53 × 10^9^
**F13: 1**	**Std.**	5.35 × 10^3^	7.79 × 10^9^	7.55 × 10^3^	9.18 × 10^9^	1.62 × 10^9^	2.32 × 10^9^	1.4 × 10^5^	5.72 × 10^9^	5.99 × 10^8^
	**Avg.**	6.06 × 10^3^	2.4 × 10^10^	1.38 × 10^4^	1.55 × 10^10^	1.4 × 10^9^	5.19 × 10^9^	2.32 × 10^5^	2.53 × 10^10^	2.32 × 10^8^
**F14: 1**	**Std.**	4.33 × 10^1^	3.52 × 10^7^	2.41 × 10^4^	1.95 × 10^7^	1.52 × 10^6^	3.7 × 10^6^	2.09 × 10^5^	1.21 × 10^7^	3.21 × 10^6^
	**Avg.**	1.65 × 10^3^	5.29 × 10^7^	2.06 × 10^4^	2.52 × 10^7^	2.29 × 10^6^	7.1 × 10^6^	4.12 × 10^5^	3.19 × 10^7^	1.89 × 10^6^
**F15: 1**	**Std.**	7.58 × 10^3^	2.23 × 10^9^	8.14 × 10^3^	3.59 × 10^9^	5.53 × 10^7^	3.23 × 10^8^	3.71 × 10^4^	2.2 × 10^9^	2.14 × 10^4^
	**Avg.**	6.11 × 10^3^	4.26 × 10^9^	1.08 × 10^4^	4.59 × 10^9^	7.33 × 10^7^	7.97 × 10^8^	6.32 × 10^4^	7.92 × 10^9^	4.38 × 10^4^
**F16: 2**	**Std.**	3.64 × 10^2^	8.45 × 10^2^	3.86 × 10^2^	9.75 × 10^2^	6 × 10^2^	3.06 × 10^2^	4.08 × 10^2^	4.68 × 10^2^	5.17 × 10^2^
	**Avg.**	3.44 × 10^3^	7.07 × 10^3^	2.8 × 10^3^	7.88 × 10^3^	4.44 × 10^3^	6.15 × 10^3^	3.57 × 10^3^	8.64 × 10^3^	4.18 × 10^3^
**F17: 2**	**Std.**	1.56 × 10^2^	6.93 × 10^2^	2.27 × 10^2^	9.93 × 10^4^	3.56 × 10^2^	2.68 × 10^2^	2.83 × 10^2^	7.24 × 10^3^	4.85 × 10^3^
	**Avg.**	2.87 × 10^3^	4.69 × 10^3^	2.65 × 10^3^	4.34 × 10^4^	3.75 × 10^3^	4.85 × 10^3^	3.28 × 10^3^	1.62 × 10^4^	4.86 × 10^3^
**F18: 1**	**Std.**	1.14 × 10^4^	3.92 × 10^7^	1.54 × 10^5^	9.29 × 10^7^	5.51 × 10^6^	2.26 × 10^7^	2.27 × 10^6^	5.21 × 10^7^	3.87 × 10^6^
	**Avg.**	7.99 × 10^3^	7.79 × 10^7^	1.45 × 10^5^	1.32 × 10^8^	8.54 × 10^6^	3.65 × 10^7^	3.36 × 10^6^	1.66 × 10^8^	5.78 × 10^6^
**F19: 1**	**Std.**	2.25 × 10^1^	7.87 × 10^8^	1.16 × 10^4^	1.21 × 10^9^	1.72 × 10^8^	2.93 × 10^8^	4.71 × 10^4^	7.5 × 10^8^	8.64 × 10^4^
	**Avg.**	2.04 × 10^3^	1.21 × 10^9^	1.73 × 10^4^	1.43 × 10^9^	7.43 × 10^7^	5.29 × 10^8^	6.56 × 10^4^	3.2 × 10^9^	5.99 × 10^4^
**F20: 2**	**Std.**	1.79 × 10^2^	2.88 × 10^2^	2.79 × 10^2^	2.75 × 10^2^	3.88 × 10^2^	1.57 × 10^2^	3.26 × 10^2^	1.38 × 10^2^	4.36 × 10^2^
	**Avg.**	3.07 × 10^3^	3.65 × 10^3^	2.8 × 10^3^	4.9 × 10^3^	3.8 × 10^3^	4.19 × 10^3^	3.2 × 10^3^	4.44 × 10^3^	3.82 × 10^3^
**F21: 3**	**Std.**	5.35 × 10^1^	5.39 × 10^1^	4.24 × 10^1^	1.36 × 10^2^	5.05 × 10^1^	3.95 × 10^1^	4.54 × 10^1^	4.6 × 10^1^	9.81 × 10^1^
	**Avg.**	2.59 × 10^3^	2.97 × 10^3^	2.52 × 10^3^	3.22 × 10^3^	2.76 × 10^3^	2.94 × 10^3^	2.52 × 10^3^	3.25 × 10^3^	2.91 × 10^3^
**F22: 4**	**Std.**	2.19 × 10^3^	8.76 × 10^2^	2.29 × 10^3^	5.33 × 10^2^	1.15 × 10^3^	4.58 × 10^2^	9.53 × 10^2^	6.11 × 10^2^	7.37 × 10^2^
	**Avg.**	1.36 × 10^4^	1.49 × 10^4^	1.06 × 10^4^	1.75 × 10^4^	1.41 × 10^4^	1.68 × 10^4^	9.24 × 10^3^	1.73 × 10^4^	1.04 × 10^4^
**F23: 1**	**Std.**	7.47 × 10^1^	2.28 × 10^2^	9.11 × 10^1^	2.03 × 10^2^	7.15 × 10^1^	7.34 × 10^1^	5.97 × 10^1^	1.09 × 10^2^	1.37 × 10^2^
	**Avg.**	3 × 10^3^	4.53 × 10^3^	3.1 × 10^3^	3.87 × 10^3^	3.23 × 10^3^	3.67 × 10^3^	3.01 × 10^3^	4.31 × 10^3^	3.64 × 10^3^
**F24: 2**	**Std.**	4.41 × 10^1^	2.44 × 10^2^	7.68 × 10^1^	1.57 × 10^2^	7.23 × 10^1^	7.67 × 10^1^	5.06 × 10^1^	1.23 × 10^2^	1.73 × 10^2^
	**Avg.**	3.23 × 10^3^	4.94 × 10^3^	3.28 × 10^3^	4.03 × 10^3^	3.33 × 10^3^	3.83 × 10^3^	3.16 × 10^3^	4.66 × 10^3^	3.75 × 10^3^
**F25: 1**	**Std.**	3.02 × 10^1^	1.13 × 10^3^	4.35 × 10^1^	9.11 × 10^3^	7.6 × 10^2^	8.72 × 10^2^	3.2 × 10^1^	3.34 × 10^3^	8.74 × 10^2^
	**Avg.**	3.06 × 10^3^	1.19 × 10^4^	3.15 × 10^3^	2.15 × 10^4^	5.84 × 10^3^	8.27 × 10^3^	3.1 × 10^3^	3.23 × 10^4^	4.23 × 10^3^
**F26: 1**	**Std.**	1.62 × 10^3^	7.66 × 10^2^	1.36 × 10^3^	2.22 × 10^3^	5.3 × 10^2^	7.53 × 10^2^	7.11 × 10^2^	1.24 × 10^3^	1.61 × 10^3^
	**Avg.**	5.52 × 10^3^	1.57 × 10^4^	7.93 × 10^3^	1.68 × 10^4^	8.65 × 10^3^	1.34 × 10^4^	6.69 × 10^3^	2.07 × 10^4^	1.37 × 10^4^
**F27: 1**	**Std.**	1.26 × 10^2^	6.3 × 10^2^	1.36 × 10^2^	3.86 × 10^2^	1.71 × 10^2^	2.19 × 10^2^	8.71 × 10^1^	3.08 × 10^2^	2.48 × 10^2^
	**Avg.**	3.37 × 10^3^	6.98 × 10^3^	3.66 × 10^3^	4.98 × 10^3^	3.94 × 10^3^	4.79 × 10^3^	3.58 × 10^3^	6.26 × 10^3^	4.08 × 10^3^
**F28: 1**	**Std.**	2.14 × 10^1^	7.57 × 10^2^	1.19 × 10^2^	2.32 × 10^3^	1.57 × 10^3^	8.55 × 10^2^	4.19 × 10^1^	1.1 × 10^3^	1.47 × 10^3^
	**Avg.**	3.3 × 10^3^	1.05 × 10^4^	3.49 × 10^3^	1.28 × 10^4^	9.17 × 10^3^	8.19 × 10^3^	3.39 × 10^3^	1.54 × 10^4^	6.22 × 10^3^
**F29: 1**	**Std.**	2.09 × 10^2^	2 × 10^4^	4.46 × 10^2^	4.95 × 10^4^	1.59 × 10^3^	9.48 × 10^2^	4.75 × 10^2^	8.54 × 10^3^	8.33 × 10^2^
	**Avg.**	4.03 × 10^3^	2.43 × 10^4^	4.52 × 10^3^	2.6 × 10^4^	7.07 × 10^3^	8.41 × 10^3^	4.98 × 10^3^	2.33 × 10^4^	6.8 × 10^3^
**F30: 1**	**Std.**	1.19 × 10^5^	1.35 × 10^9^	8.79 × 10^5^	2.48 × 10^9^	3.68 × 10^8^	3.16 × 10^8^	7.77 × 10^6^	1.27 × 10^9^	4.57 × 10^8^
	**Avg.**	7.28 × 10^5^	2.91 × 10^9^	1.65 × 10^6^	3.67 × 10^9^	3.39 × 10^8^	1 × 10^9^	2.71 × 10^7^	5.45 × 10^9^	2.03 × 10^8^
**Average rank**		1.59 × 10^0^	6.68 × 10^0^	2.17 × 10^0^	8.1 × 10^0^	4.59 × 10^0^	6.1 × 10^0^	2.45 × 10^0^	8.48 × 10^0^	4.66 × 10^0^
Friedman Chi-square statistic = 204.533333										
Chi-square critical value (k = 9, N = 29, alpha = 0.05) = 15.507313 *p*-value = 7.079 × 10^−40^										

**Table 10:** Results for 100 dimensions on the CEC 2017 benchmark.

Func.	Metric	WDHOA	HOA	LSHADE	SFOA	STOA	SCA	RIME	KOA	CPSOGSA
**F1: 1**	**Std.**	1.46 × 10^7^	1.3 × 10^10^	4.21 × 10^9^	7.44 × 10^10^	1.27 × 10^10^	1.61 × 10^10^	4.2 × 10^7^	2.04 × 10^10^	3.6 × 10^10^
	**Avg.**	4.01 × 10^7^	2.27 × 10^11^	1.31 × 10^10^	3.51 × 10^11^	1.48 × 10^11^	2.04 × 10^11^	1.64 × 10^8^	4.72 × 10^11^	8.66 × 10^10^
**F3: 2**	**Std.**	4.36 × 10^4^	1.18 × 10^4^	2.86 × 10^4^	4.21 × 10^5^	1.16 × 10^5^	5.24 × 10^4^	9.3 × 10^4^	7.31 × 10^4^	1.48 × 10^5^
	**Avg.**	2.13 × 10^5^	3.2 × 10^5^	2 × 10^5^	1.32 × 10^6^	5.37 × 10^5^	4.71 × 10^5^	6.31 × 10^5^	7.57 × 10^5^	9.63 × 10^5^
**F4: 1**	**Std.**	5.3 × 10^1^	9.36 × 10^3^	5.54 × 10^2^	5.17 × 10^4^	4.79 × 10^3^	7.27 × 10^3^	7.64 × 10^1^	1.33 × 10^4^	5.09 × 10^3^
	**Avg.**	8.18 × 10^2^	6.85 × 10^4^	2.22 × 10^3^	1.28 × 10^5^	1.93 × 10^4^	4.64 × 10^4^	9.57 × 10^2^	1.66 × 10^5^	1.31 × 10^4^
**F5: 3**	**Std.**	7.42 × 10^1^	5.35 × 10^1^	9.52 × 10^1^	2.35 × 10^2^	8.74 × 10^1^	4.56 × 10^1^	8.1 × 10^1^	6.05 × 10^1^	1.58 × 10^2^
	**Avg.**	1.44 × 10^3^	1.91 × 10^3^	1.21 × 10^3^	2.47 × 10^3^	1.66 × 10^3^	2.03 × 10^3^	1.16 × 10^3^	2.69 × 10^3^	1.87 × 10^3^
**F6: 1**	**Std.**	2.62 × 10^0^	4.15 × 10^0^	5.92 × 10^0^	1.31 × 10^1^	5.72 × 10^0^	5.93 × 10^0^	7.05 × 10^0^	4.5 × 10^0^	4.77 × 10^0^
	**Avg.**	6.11 × 10^2^	6.97 × 10^2^	6.46 × 10^2^	7.34 × 10^2^	6.85 × 10^2^	7.03 × 10^2^	6.47 × 10^2^	7.39 × 10^2^	6.85 × 10^2^
**F7: 2**	**Std.**	9.76 × 10^1^	1.16 × 10^2^	2.58 × 10^2^	2.45 × 10^3^	1.77 × 10^2^	2.16 × 10^2^	1.91 × 10^2^	3.45 × 10^2^	7.73 × 10^2^
	**Avg.**	1.93 × 10^3^	3.67 × 10^3^	2.7 × 10^3^	7.05 × 10^3^	3.3 × 10^3^	3.87 × 10^3^	1.87 × 10^3^	1.1 × 10^4^	7.09 × 10^3^
**F8: 3**	**Std.**	8.77 × 10^1^	6.7 × 10^1^	1.23 × 10^2^	1.9 × 10^2^	8.4 × 10^1^	7.33 × 10^1^	9.29 × 10^1^	8.02 × 10^1^	1.54 × 10^2^
	**Avg.**	1.69 × 10^3^	2.39 × 10^3^	1.6 × 10^3^	2.93 × 10^3^	1.98 × 10^3^	2.37 × 10^3^	1.5 × 10^3^	3.1 × 10^3^	2.27 × 10^3^
**F9: 1**	**Std.**	1.09 × 10^4^	7 × 10^3^	5.07 × 10^3^	3.23 × 10^4^	1.02 × 10^4^	1 × 10^4^	1.58 × 10^4^	9.84 × 10^3^	5.7 × 10^3^
	**Avg.**	3.8 × 10^4^	6.55 × 10^4^	3.87 × 10^4^	1.56 × 10^5^	5.91 × 10^4^	8.53 × 10^4^	4.14 × 10^4^	1.61 × 10^5^	4.55 × 10^4^
**F10: 4**	**Std.**	5.68 × 10^2^	1.1 × 10^3^	8.76 × 10^2^	6.34 × 10^2^	2.2 × 10^3^	8.31 × 10^2^	1.66 × 10^3^	5.56 × 10^2^	1.42 × 10^3^
	**Avg.**	2.84 × 10^4^	2.9 × 10^4^	2.28 × 10^4^	3.41 × 10^4^	2.88 × 10^4^	3.26 × 10^4^	1.74 × 10^4^	3.32 × 10^4^	1.71 × 10^4^
**F11: 1**	**Std.**	6.36 × 10^2^	3.9 × 10^4^	5.01 × 10^3^	2.17 × 10^5^	2.06 × 10^4^	2.82 × 10^4^	2.87 × 10^3^	5.19 × 10^4^	7.47 × 10^4^
	**Avg.**	3.76 × 10^3^	1.74 × 10^5^	1.19 × 10^4^	5.65 × 10^5^	9.86 × 10^4^	1.49 × 10^5^	1.33 × 10^4^	3.37 × 10^5^	2.49 × 10^5^
**F12: 1**	**Std.**	2.02 × 10^7^	2.02 × 10^10^	3.98 × 10^8^	3.78 × 10^10^	9.4 × 10^9^	1.07 × 10^10^	3.09 × 10^8^	1.4 × 10^10^	1.27 × 10^10^
	**Avg.**	2.66 × 10^7^	1.48 × 10^11^	5.87 × 10^8^	1.54 × 10^11^	3.8 × 10^10^	9.17 × 10^10^	8.17 × 10^8^	2.34 × 10^11^	1.4 × 10^10^
**F13: 1**	**Std.**	1.39 × 10^4^	5.75 × 10^9^	5.53 × 10^5^	1.94 × 10^10^	2.39 × 10^9^	3.14 × 10^9^	1.94 × 10^5^	5.47 × 10^9^	3.06 × 10^9^
	**Avg.**	1.07 × 10^4^	3.34 × 10^10^	1.7 × 10^5^	3.79 × 10^10^	6.03 × 10^9^	1.57 × 10^10^	4.63 × 10^5^	5.2 × 10^10^	9.05 × 10^8^
**F14: 1**	**Std.**	2.29 × 10^4^	2.04 × 10^7^	3.35 × 10^5^	8.81 × 10^7^	6.76 × 10^6^	2.67 × 10^7^	2.64 × 10^6^	4.43 × 10^7^	8.49 × 10^6^
	**Avg.**	1.24 × 10^4^	3.98 × 10^7^	4.34 × 10^5^	1.56 × 10^8^	1.21 × 10^7^	5.24 × 10^7^	4.7 × 10^6^	1.85 × 10^8^	1.32 × 10^7^
**F15: 1**	**Std.**	2.57 × 10^3^	4.43 × 10^9^	5.79 × 10^3^	8.65 × 10^9^	1.04 × 10^9^	1.28 × 10^9^	1.52 × 10^5^	4.04 × 10^9^	9.29 × 10^8^
	**Avg.**	4.1 × 10^3^	1.65 × 10^10^	1.09 × 10^4^	1.69 × 10^10^	1.58 × 10^9^	4.94 × 10^9^	1.66 × 10^5^	2.27 × 10^10^	2.54 × 10^8^
**F16: 3**	**Std.**	1.24 × 10^3^	2.27 × 10^3^	7.02 × 10^2^	3.34 × 10^3^	9.57 × 10^2^	6.13 × 10^2^	6.96 × 10^2^	1.74 × 10^3^	1.21 × 10^3^
	**Avg.**	7.92 × 10^3^	1.79 × 10^4^	5.38 × 10^3^	1.93 × 10^4^	9.44 × 10^3^	1.44 × 10^4^	6.93 × 10^3^	2.38 × 10^4^	8.18 × 10^3^
**F17: 3**	**Std.**	4.61 × 10^2^	2.02 × 10^6^	5.28 × 10^2^	5.7 × 10^6^	2.63 × 10^3^	2.96 × 10^4^	6.39 × 10^2^	1.67 × 10^6^	9.85 × 10^3^
	**Avg.**	6.09 × 10^3^	2.41 × 10^6^	5.27 × 10^3^	2.6 × 10^6^	8.25 × 10^3^	4.24 × 10^4^	5.81 × 10^3^	3.13 × 10^6^	9.36 × 10^3^
**F18: 1**	**Std.**	6.16 × 10^4^	2.69 × 10^7^	3.31 × 10^5^	1.74 × 10^8^	5.1 × 10^6^	4.84 × 10^7^	3.06 × 10^6^	8.14 × 10^7^	9.78 × 10^6^
	**Avg.**	1.42 × 10^5^	5.59 × 10^7^	6.49 × 10^5^	2.85 × 10^8^	1.23 × 10^7^	9.76 × 10^7^	7.22 × 10^6^	3.28 × 10^8^	1.43 × 10^7^
**F19: 1**	**Std.**	4.96 × 10^3^	3.64 × 10^9^	3.91 × 10^4^	7.12 × 10^9^	1.26 × 10^9^	9.19 × 10^8^	7.01 × 10^6^	3.1 × 10^9^	2.48 × 10^8^
	**Avg.**	6.07 × 10^3^	1.46 × 10^10^	2.24 × 10^4^	1.45 × 10^10^	1.58 × 10^9^	4.16 × 10^9^	1.25 × 10^7^	2.36 × 10^10^	7.91 × 10^7^
**F20: 4**	**Std.**	3.77 × 10^2^	4.43 × 10^2^	6.02 × 10^2^	3.25 × 10^2^	8.65 × 10^2^	3.02 × 10^2^	5.62 × 10^2^	2.46 × 10^2^	6.22 × 10^2^
	**Avg.**	6.33 × 10^3^	6.82 × 10^3^	5.1 × 10^3^	8.94 × 10^3^	6.61 × 10^3^	7.85 × 10^3^	5.69 × 10^3^	8.17 × 10^3^	6.21 × 10^3^
**F21: 2**	**Std.**	1.05 × 10^2^	1.57 × 10^2^	1.46 × 10^2^	1.88 × 10^2^	1.12 × 10^2^	8.43 × 10^1^	9.09 × 10^1^	8.15 × 10^1^	1.68 × 10^2^
	**Avg.**	3.17 × 10^3^	4.39 × 10^3^	3.19 × 10^3^	4.69 × 10^3^	3.63 × 10^3^	4.15 × 10^3^	3 × 10^3^	4.84 × 10^3^	4.04 × 10^3^
**F22: 5**	**Std.**	7.4 × 10^2^	1.25 × 10^3^	7.98 × 10^2^	6.15 × 10^2^	1.93 × 10^3^	6.87 × 10^2^	1.52 × 10^3^	6.9 × 10^2^	1.13 × 10^3^
	**Avg.**	3.04 × 10^4^	3.2 × 10^4^	2.54 × 10^4^	3.58 × 10^4^	3.03 × 10^4^	3.5 × 10^4^	1.97 × 10^4^	3.56 × 10^4^	1.97 × 10^4^
**F23: 1**	**Std.**	9.77 × 10^1^	4.02 × 10^2^	2.24 × 10^2^	3.21 × 10^2^	1.19 × 10^2^	1.37 × 10^2^	8.65 × 10^1^	2.15 × 10^2^	1.97 × 10^2^
	**Avg.**	3.38 × 10^3^	7.25 × 10^3^	4.09 × 10^3^	5.42 × 10^3^	4.12 × 10^3^	5.15 × 10^3^	3.56 × 10^3^	6.51 × 10^3^	4.81 × 10^3^
**F24: 1**	**Std.**	1.3 × 10^2^	5.9 × 10^2^	3.61 × 10^2^	6.83 × 10^2^	1.13 × 10^2^	3.57 × 10^2^	1.52 × 10^2^	4.34 × 10^2^	4.44 × 10^2^
	**Avg.**	4.01 × 10^3^	1.2 × 10^4^	5.12 × 10^3^	7.31 × 10^3^	4.92 × 10^3^	7.2 × 10^3^	4.25 × 10^3^	1.05 × 10^4^	6.31 × 10^3^
**F25: 1**	**Std.**	6.43 × 10^1^	2.33 × 10^3^	2.69 × 10^2^	2.31 × 10^4^	2.92 × 10^3^	2.51 × 10^3^	7.42 × 10^1^	7.94 × 10^3^	3.3 × 10^3^
	**Avg.**	3.51 × 10^3^	2.43 × 10^4^	4.43 × 10^3^	5.34 × 10^4^	1.33 × 10^4^	2 × 10^4^	3.69 × 10^3^	8.55 × 10^4^	1.19 × 10^4^
**F26: 1**	**Std.**	3.76 × 10^3^	2.89 × 10^3^	3.36 × 10^3^	5.89 × 10^3^	1.38 × 10^3^	2.4 × 10^3^	1.49 × 10^3^	4.12 × 10^3^	3.97 × 10^3^
	**Avg.**	1.08 × 10^4^	4.91 × 10^4^	2.37 × 10^4^	4.7 × 10^4^	2.21 × 10^4^	3.96 × 10^4^	1.5 × 10^4^	6.34 × 10^4^	3.74 × 10^4^
**F27: 1**	**Std.**	6.16 × 10^1^	1.22 × 10^3^	3.49 × 10^2^	1.47 × 10^3^	3.55 × 10^2^	4.88 × 10^2^	1.29 × 10^2^	9.58 × 10^2^	3.94 × 10^2^
	**Avg.**	3.5 × 10^3^	1.27 × 10^4^	4.31 × 10^3^	8.73 × 10^3^	4.86 × 10^3^	8.26 × 10^3^	3.92 × 10^3^	1.18 × 10^4^	4.94 × 10^3^
**F28: 1**	**Std.**	5.08 × 10^1^	2.04 × 10^3^	9.6 × 10^2^	8.58 × 10^3^	5.47 × 10^3^	2.12 × 10^3^	7 × 10^1^	2.86 × 10^3^	4.63 × 10^3^
	**Avg.**	3.55 × 10^3^	3.07 × 10^4^	5.99 × 10^3^	4.36 × 10^4^	2.46 × 10^4^	2.56 × 10^4^	3.78 × 10^3^	5.28 × 10^4^	2.05 × 10^4^
**F29: 1**	**Std.**	7.62 × 10^2^	7.64 × 10^4^	7.31 × 10^2^	1.56 × 10^6^	5.4 × 10^3^	1.02 × 10^4^	9.46 × 10^2^	3.81 × 10^5^	1.78 × 10^3^
	**Avg.**	7.61 × 10^3^	1.2 × 10^5^	8.37 × 10^3^	6.88 × 10^5^	1.58 × 10^4^	3.12 × 10^4^	9.47 × 10^3^	7.11 × 10^5^	1.31 × 10^4^
**F30: 1**	**Std.**	1.85 × 10^4^	5.4 × 10^9^	1.57 × 10^6^	1.5 × 10^10^	1.29 × 10^9^	2.49 × 10^9^	5.19 × 10^7^	5.08 × 10^9^	6.76 × 10^8^
	**Avg.**	2.94 × 10^4^	2.66 × 10^10^	2.02 × 10^6^	2.86 × 10^10^	3.43 × 10^9^	1.23 × 10^10^	1.11 × 10^8^	3.69 × 10^10^	3.91 × 10^8^
**Average rank**		1.72 × 10^0^	6.76 × 10^0^	2.34 × 10^0^	7.97 × 10^0^	4.48 × 10^0^	6.17 × 10^0^	2.38 × 10^0^	8.69 × 10^0^	4.48 × 10^0^
Friedman Chi-square statistic = 201.296552										
Chi-square critical value (k = 9, N = 29, alpha = 0.05) = 15.507313 *p*-value = 3.406 × 10^−39^										

**Table 11:** Detailed configurations of the HOA variants.

Strategy	WDHOA	HOA	AHOA	BHOA	CHOA	DHOA	EHOA	FHOA
**Dynamic Grouping**	YES	NO	NO	YES	YES	NO	YES	NO
**Hybrid Search**	YES	NO	YES	NO	YES	NO	NO	YES
**Angle Generation**	YES	NO	YES	YES	NO	YES	NO	NO

**Table 12:** Results for ablation experiment on the CEC 2022 benchmark.

Func.	Metric	WDHOA	HOA	AHOA	BHOA	CHOA	DHOA	EHOA	FHOA
**F1**	**Std.**	0	2.81 × 10^3^	2.17 × 10^2^	1.06 × 10^−14^	0	6.18 × 10^2^	0	1.83 × 10^3^
	**Avg.**	3 × 10^2^	7.03 × 10^3^	5.42 × 10^2^	3 × 10^2^	3 × 10^2^	1.02 × 10^3^	3 × 10^2^	3.04 × 10^3^
	**Rank**	1 × 10^0^	8 × 10^0^	5 × 10^0^	4 × 10^0^	1 × 10^0^	6 × 10^0^	1 × 10^0^	7 × 10^0^
**F2**	**Std.**	3.76 × 10^0^	2.17 × 10^2^	1.31 × 10^1^	3.56 × 10^0^	2.41 × 10^1^	1.46 × 10^1^	2.13 × 10^1^	3.14 × 10^1^
	**Avg.**	4.04 × 10^2^	7.21 × 10^2^	4.06 × 10^2^	4.03 × 10^2^	4.11 × 10^2^	4.09 × 10^2^	4.08 × 10^2^	4.26 × 10^2^
	**Rank**	2 × 10^0^	8 × 10^0^	3 × 10^0^	1 × 10^0^	6 × 10^0^	5 × 10^0^	4 × 10^0^	7 × 10^0^
**F3**	**Std.**	1.3 × 10^−5^	9.09 × 10^0^	1.9 × 10^−1^	1.51 × 10^−3^	4.98 × 10^−3^	1.31 × 10^0^	4.32 × 10^0^	9.13 × 10^−1^
	**Avg.**	6 × 10^2^	6.3 × 10^2^	6 × 10^2^	6 × 10^2^	6 × 10^2^	6.01 × 10^2^	6.04 × 10^2^	6.01 × 10^2^
	**Rank**	1 × 10^0^	8 × 10^0^	4 × 10^0^	2 × 10^0^	3 × 10^0^	5 × 10^0^	7 × 10^0^	6 × 10^0^
**F4**	**Std.**	3.16 × 10^0^	8.63 × 10^0^	3.67 × 10^0^	4.47 × 10^0^	4.15 × 10^0^	5.33 × 10^0^	7.59 × 10^0^	7.11 × 10^0^
	**Avg.**	8.1 × 10^2^	8.33 × 10^2^	8.12 × 10^2^	8.13 × 10^2^	8.13 × 10^2^	8.14 × 10^2^	8.19 × 10^2^	8.2 × 10^2^
	**Rank**	1 × 10^0^	8 × 10^0^	2 × 10^0^	4 × 10^0^	3 × 10^0^	5 × 10^0^	6 × 10^0^	7 × 10^0^
**F5**	**Std.**	8.57 × 10^−2^	1.26 × 10^2^	1.23 × 10^0^	2.6 × 10^−1^	1.35 × 10^0^	9.55 × 10^0^	3.97 × 10^1^	3.9 × 10^1^
	**Avg.**	9 × 10^2^	1.12 × 10^3^	9.01 × 10^2^	9 × 10^2^	9.01 × 10^2^	9.04 × 10^2^	9.21 × 10^2^	9.58 × 10^2^
	**Rank**	1 × 10^0^	8 × 10^0^	4 × 10^0^	2 × 10^0^	3 × 10^0^	5 × 10^0^	6 × 10^0^	7 × 10^0^
**F6**	**Std.**	5.28 × 10^−1^	1.26 × 10^8^	1.19 × 10^3^	6.48 × 10^0^	5.15 × 10^−1^	1.99 × 10^3^	1.65 × 10^1^	4.53 × 10^5^
	**Avg.**	1.8 × 10^3^	6.88 × 10^7^	2.83 × 10^3^	1.8 × 10^3^	1.8 × 10^3^	3.72 × 10^3^	1.81 × 10^3^	1.35 × 10^5^
	**Rank**	2 × 10^0^	8 × 10^0^	5 × 10^0^	3 × 10^0^	1 × 10^0^	6 × 10^0^	4 × 10^0^	7 × 10^0^
**F7**	**Std.**	6.36 × 10^0^	1.72 × 10^1^	7.69 × 10^0^	8.77 × 10^0^	9.81 × 10^0^	8.55 × 10^0^	1.16 × 10^1^	9.2 × 10^0^
	**Avg.**	2 × 10^3^	2.07 × 10^3^	2.01 × 10^3^	2.02 × 10^3^	2.01 × 10^3^	2.02 × 10^3^	2.03 × 10^3^	2.01 × 10^3^
	**Rank**	1 × 10^0^	8 × 10^0^	2 × 10^0^	5 × 10^0^	3 × 10^0^	6 × 10^0^	7 × 10^0^	4 × 10^0^
**F8**	**Std.**	7.2 × 10^0^	3.79 × 10^1^	6.3 × 10^0^	6.89 × 10^0^	9.24 × 10^0^	3.66 × 10^0^	2.27 × 10^1^	3.06 × 10^0^
	**Avg.**	2.21 × 10^3^	2.24 × 10^3^	2.22 × 10^3^	2.22 × 10^3^	2.21 × 10^3^	2.22 × 10^3^	2.22 × 10^3^	2.22 × 10^3^
	**Rank**	1 × 10^0^	8 × 10^0^	3 × 10^0^	4 × 10^0^	2 × 10^0^	7 × 10^0^	6 × 10^0^	5 × 10^0^
**F9**	**Std.**	0	3.02 × 10^1^	2.65 × 10^−3^	8.44 × 10^−14^	0	8.69 × 10^−8^	2.68 × 10^1^	1.62 × 10^1^
	**Avg.**	2.53 × 10^3^	2.69 × 10^3^	2.53 × 10^3^	2.53 × 10^3^	2.53 × 10^3^	2.53 × 10^3^	2.53 × 10^3^	2.58 × 10^3^
	**Rank**	1 × 10^0^	8 × 10^0^	5 × 10^0^	3 × 10^0^	1 × 10^0^	4 × 10^0^	6 × 10^0^	7 × 10^0^
**F10**	**Std.**	2.8 × 10^1^	5.22 × 10^1^	3.86 × 10^1^	3.47 × 10^1^	5.82 × 10^1^	3.94 × 10^1^	6.09 × 10^1^	6.34 × 10^1^
	**Avg.**	2.51 × 10^3^	2.64 × 10^3^	2.51 × 10^3^	2.51 × 10^3^	2.55 × 10^3^	2.52 × 10^3^	2.56 × 10^3^	2.55 × 10^3^
	**Rank**	1 × 10^0^	8 × 10^0^	3 × 10^0^	2 × 10^0^	5 × 10^0^	4 × 10^0^	7 × 10^0^	6 × 10^0^
**F11**	**Std.**	1.05 × 10^2^	4.33 × 10^2^	1.47 × 10^2^	1.22 × 10^2^	1.32 × 10^2^	1.57 × 10^2^	1.44 × 10^2^	1.8 × 10^2^
	**Avg.**	2.65 × 10^3^	3.39 × 10^3^	2.8 × 10^3^	2.66 × 10^3^	2.83 × 10^3^	2.73 × 10^3^	2.78 × 10^3^	2.94 × 10^3^
	**Rank**	1 × 10^0^	8 × 10^0^	5 × 10^0^	2 × 10^0^	6 × 10^0^	3 × 10^0^	4 × 10^0^	7 × 10^0^
**F12**	**Std.**	1.35 × 10^0^	4.92 × 10^1^	1.56 × 10^0^	3.01 × 10^0^	1.65 × 10^0^	1.6 × 10^0^	1.6 × 10^1^	2.83 × 10^1^
	**Avg.**	2.86 × 10^3^	3.01 × 10^3^	2.86 × 10^3^	2.86 × 10^3^	2.86 × 10^3^	2.86 × 10^3^	2.87 × 10^3^	2.9 × 10^3^
	**Rank**	1 × 10^0^	8 × 10^0^	2 × 10^0^	3 × 10^0^	4 × 10^0^	5 × 10^0^	6 × 10^0^	7 × 10^0^
**Average rank**		1.17 × 10^0^	8 × 10^0^	3.58 × 10^0^	2.92 × 10^0^	3.17 × 10^0^	5.08 × 10^0^	5.33 × 10^0^	6.42 × 10^0^
Friedman Chi-square statistic = 65.436620									
Chi-square critical value (k=8, N=12, alpha=0.05) = 14.067143 *p*-value = 1.229 × 10^−11^									

**Table 13:** Weight settings of global fitness function.

**Weight**	**Number**	**Meaning**
Collision weight $\omega_{c}$	1000	Physical absolute constraints
Distance weight $\omega_{d}$	1	Evacuation path length
Turning penalty weight $\omega_{t}$	1.5	Maintain path smoothness

**Table 14:** Summary of single-source global fitness function evaluation metrics.

Algorithm	Path Length	Turning Penalty	Collision Cost	Total Cost
**WDHOA**	**39.21**	**0**	**0**	**39.21**
**HOA**	45.46	3	90	90,049.96
**LSHADE**	41.21	1	0	42.71
**SFOA**	48.97	5	200	200,056.47
**STOA**	65.21	5	150	150,072.71
**SCA**	53.46	6	150	150,062.46
**RIME**	42.63	1	0	44.13
**KOA**	39.8	0	0	39.80
**CPSOGSA**	61.7	3	60	60,066.20

**Table 15:** Summary of multi-source global fitness function evaluation metrics.

Algorithm	Path Length	Turning Penalty	Collision Cost	Total Cost
**WDHOA**	**38.21**	**0**	**0**	**38.21**
**HOA**	41.53	4	100	100,047.53
**LSHADE**	39.97	3	0	44.47
**SFOA**	46.41	1	300	300,047.91
**STOA**	61.38	4	150	150,067.38
**SCA**	63.28	5	110	110,070.78
**RIME**	40.46	1	0	41.96
**KOA**	40.46	3	0	44.96
**CPSOGSA**	45.87	2	50	50,048.87

## Data Availability

Data is contained within the article.
